# Supplementary material for: STOP leg clots—Swedish multicentre trial of outpatient prevention of leg clots: study protocol for a randomised controlled trial on the efficacy of intermittent pneumatic compression on venous thromboembolism in lower leg immobilised patients
Source: BMJ Open. 2021 May 20;11(5):e044103. doi: 10.1136/bmjopen-2020-044103 (PMC8141452; doi:10.1136/bmjopen-2020-044103)
Supplement: Author's manuscript [file bmjopen-2020-044103.draft_revisions.pdf]

BMJ Open

BMJ Open is committed to open peer review. As part of this commitment we make the peer review history of every article we publish publicly available.

When an article is published we post the peer reviewers' comments and the authors' responses online. We also post the versions of the paper that were used during peer review. These are the versions that the peer review comments apply to.

The versions of the paper that follow are the versions that were submitted during the peer review process. They are not the versions of record or the final published versions. They should not be cited or distributed as the published version of this manuscript.

BMJ Open is an open access journal and the full, final, typeset and author-corrected version of record of the manuscript is available on our site with no access controls, subscription charges or pay-per-view fees (http://bmjopen.bmj.com).

If you have any questions on BMJ Open's open peer review process please email info.bmjopen@bmj.com

BMJ Open

STOP Leg Clots - Swedish multicenter Trial of Outpatient Prevention of Leg Clots: study protocol for a randomized controlled trial on the efficacy of intermittent pneumatic compression on venous thromboembolism in lower leg immobilized patients

Journal:	*BMJ Open*
Manuscript ID	bmjopen-2020-044103
Article Type:	Protocol
Date Submitted by the Author:	28-Sep-2020

Complete List of Authors:	Svedman, Simon; Karolinska Institutet, Molecular Medicine and Surgery; Karolinska University Hospital, Department of Orthopedic Surgery<br>Alkner, Björn; Linköping University, Department of Orthopaedics, Eksjö, Region Jönköping County and Department of Biomedical Clinical Science<br>Berg, Hans; Karolinska University Hospital, Department of Orthopedic Surgery; Karolinska institutet Department of Clinical Sciences Intervention and Technology, Division of Orthopaedics and Biotechnology<br>Domeij-Arverud, Erica; Karolinska Institutet, Department och clinical sciences, Danderyd Hospital<br>Jonsson, Kenneth; Uppsala University Hospital, Department of Surgical Sciences<br>Nilsson Helander, Katarina; Sahlgrenska University Hospital, Department of Orhopaedics<br>Ackermann, Paul; Karolinska Institutet, Department of Molecular Medicine and Surgery; Karolinska University Hospital, Department of Orthopedic Surgery

Keywords:	Foot & ankle < ORTHOPAEDIC & TRAUMA SURGERY, Thromboembolism < CARDIOLOGY, Anticoagulation < HAEMATOLOGY, Orthopaedic sports trauma < ORTHOPAEDIC & TRAUMA SURGERY, Ultrasound < RADIOLOGY & IMAGING, Orthopaedic & trauma surgery < SURGERY

SCHOLARONE™
Manuscripts

I, the Submitting Author has the right to grant and does grant on behalf of all authors of the Work (as defined in the below author licence), an exclusive licence and/or a non-exclusive licence for contributions from authors who are: i) UK Crown employees; ii) where BMJ has agreed a CC-BY licence shall apply, and/or iii) in accordance with the terms applicable for US Federal Government officers or employees acting as part of their official duties; on a worldwide, perpetual, irrevocable, royalty-free basis to BMJ Publishing Group Ltd ("BMJ") its licensees and where the relevant Journal is co-owned by BMJ to the co-owners of the Journal, to publish the Work in this journal and any other BMJ products and to exploit all rights, as set out in our licence.

The Submitting Author accepts and understands that any supply made under these terms is made by BMJ to the Submitting Author unless you are acting as an employee on behalf of your employer or a postgraduate student of an affiliated institution which is paying any applicable article publishing charge ("APC") for Open Access articles. Where the Submitting Author wishes to make the Work available on an Open Access basis (and intends to pay the relevant APC), the terms of reuse of such Open Access shall be governed by a Creative Commons licence – details of these licences and which Creative Commons licence will apply to this Work are set out in our licence referred to above.

Other than as permitted in any relevant BMJ Author's Self Archiving Policies, I confirm this Work has not been accepted for publication elsewhere, is not being considered for publication elsewhere and does not duplicate material already published. I confirm all authors consent to publication of this Work and authorise the granting of this licence.

For peer review only

STOP Leg Clots - Swedish multicenter Trial of Outpatient Prevention of Leg Clots: study protocol for a randomized controlled trial on the efficacy of intermittent pneumatic compression on venous thromboembolism in lower leg immobilized patients

Simon Svedman[1,2], Björn A. Alkner[3], Hans E Berg[2,4], Erica Domeij-Arverud[5], Kenneth B. Jonsson[6], Katarina Nilsson Helander[7], Paul W. Ackermann[1,2]

1 - Department of Molecular Medicine and Surgery, Karolinska Institutet, Stockholm, Sweden.

2 - Department of Orthopedic Surgery, Karolinska University Hospital, Stockholm, Sweden

3 - Department of Orthopaedics, Eksjö, Region Jönköping County and Department of Biomedical Clinical Science, Linköping University, Linköping, Sweden

4 - Division of Orthopaedics and Biotechnology, Department of Clinical Science, Intervention and Technology, Karolinska Institutet, Stockholm, Sweden

5 - Karolinska Institutet, Department of Clinical Sciences, Danderyd Hospital, Stockholm, Sweden.

6 - Department of Surgical Sciences, Uppsala University Hospital, Uppsala, Sweden

7 - Department of Orthopaedics, Sahlgrenska University Hospital, Gothenburg, Sweden

Email

Simon.svedman@ki.se, bjorn.alkner@liu.se, hans.er.berg@sll.se, Erica.domeij-arverud@sll.se, kenneth.jonsson@surgsci.uu.se, katarina.nilsson.helander@vgregion.se, Paul.ackermann@ki.se

Corresponding author:

Paul W. Ackermann; Paul.ackermann@ki.se

For peer review only - http://bmjopen.bmj.com/site/about/guidelines.xhtml

Abstract:

Introduction:

Leg immobilization in a cast or an orthosis after lower limb injuries is associated with a high risk of complications of venous thromboembolism (VTE) and hampered healing. Current pharmacoprophylaxes of VTE are inefficient and associated with adverse events. Intermittent pneumatic compression (IPC could represent a novel, efficient and safe VTE-prophylactic alternative that may enhance injury healing. The aim of STOP leg clots is to demonstrate that adjuvant IPC-therapy reduces VTE-incidence and improves healing in lower leg immobilized outpatients.

Methods and analysis:

STOP leg clots is a multicentre randomised controlled superiority trial conducted across Sweden. Eligible patients (700 patients/arm) with either an acute ankle fracture or Achilles tendon rupture will be randomized to either IPC during the immobilization period or treatment-as-usual. The primary outcome will be the total VTE-incidence during the leg immobilization period, approximately 6-8 weeks. VTE-incidence will be assessed by screening compression duplex ultrasound at removal of leg immobilization and/or clinically diagnosed pulmonary embolisms verified by computer tomography.

Secondary outcomes will include patient-reported outcome using validated questionnaires, healing evaluated by measurements of tendon callus production and changes in VTE-prophylactic mechanisms assessed by blood flow and profibrinolytic substances. Data analyses will be blinded and based on the intention-to-treat.

For peer review only

For peer review only - http://bmjopen.bmj.com/site/about/guidelines.xhtml

Ethics and dissemination:

Ethical approval was obtained by the ethical review board in Stockholm, Sweden, Dnr 2016/1573-31. The study will be conducted in accordance with the Helsinki declaration. The results of the study will be disseminated in peer-reviewed international journals.

Trial Registration:

ClinicalTrials.gov Identifier: NCT03259204, registered August 23, 2017.

Strengths and limitations of this study

- This is the first multicentre randomized controlled trial to investigate the efficiency of adjuvant IPC on VTE for the duration of ankle joint immobilization in an outpatient setting.
- The multicenter design improves the external validity of the study and will allow rapid dissemination and potential implementation of study results.
- Patients cannot be blinded to allocation.
- Clinical sonographers are responsible to assess the primary outcome and will be blinded to patient allocation.
- The duplex ultrasound screening will detect subclinical VTE which will allow high study power. However, the clinical relevance of non-symptomatic events is still not fully clear.

For peer review only

For peer review only - http://bmjopen.bmj.com/site/about/guidelines.xhtml

Introduction

Venous thromboembolism (VTE), comprising deep venous thrombosis (DVT) and pulmonary embolism (PE), is a dangerous medical condition. Leg immobilization is a common cause of VTE due to venous stasis (1). Prolonged leg immobilization, approximately 6-8 weeks, is a general treatment of patients with injuries and surgery to the lower limbs, which may lead to complications of muscle atrophy and hampered healing in addition to VTE. Estimated DVT incidence may range from 21% up to 50% after ankle fractures (AF) and Achilles tendon ruptures (ATR), respectively (2, 3).

The most used method to prevent VTE is through pharmacological prophylaxis, such as low molecular weight heparin (LMWH). However, several studies have demonstrated only a marginal or no effect of LMWH in lower leg immobilized patients (4-8). In addition to their low efficacy, LMWH increases the risk of bleeding complications (6, 8) and may impair tissue healing (9). For these reasons, the National Health Service in UK, and the corresponding authority in Sweden, have stressed the need for safer and more efficient VTE-prophylaxis (10-12).

Mechanical thromboprophylaxis with intermittent pneumatic compression (IPC) reduces VTE, is a cost-effective intervention in hospitalized patients, and is recommended by guidelines for surgical in-hospital patients (10, 13). Whether long-term IPC treatment applied during leg immobilization in an outpatient setting can reduce the incidence of DVT is unknown.

We recently published a study presenting preliminary evidence that leg immobilized patients with ATR treated with adjuvant IPC at 2 weeks had a reduced DVT rate (21% vs. 37%) compared to controls using Doppler ultrasound screening. Moreover, our follow-up study demonstrated a 70 % increased callus production at 2 weeks in

For peer review only - http://bmjopen.bmj.com/site/about/guidelines.xhtml

the patients with adjuvant IPC. However, in the previous study the IPC intervention ended at two weeks and at 6 weeks the DVT-rate was 50% in both groups (2). Thus, the primary aim of this prospective, multicentre, randomized controlled trial is to provide reliable evidence of the efficacy of prolonged adjuvant IPC therapy in leg-immobilized patients on the total incidence of VTE up to mobilization. As recent studies suggest that the increased neuro-vascular flow induced by IPC could trigger the healing process (14), we also aim to assess whether adjuvant IPC enhances healing, reduced muscle atrophy and improves the long-term patient-reported outcomes in lower limb immobilized patients.

Methods and analysis

This is a report of the trial protocol dated 4th September 2017, supported by the Swedish research council. Any important protocol modifications will be communicated through ClinicalTrials.gov trial number NCT03259204, the Ethical Review Board and/or the BMJ Open journal. The protocol was developed in accordance with Standard Protocol Items: Recommendations for Interventional Trials and Template for Intervention Description and Replication statements (15).

Study design and setting

“STOP leg clots” is a Swedish nationwide, multicentre, prospective, superiority, randomized, controlled, parallel group trial with blinded assessment of the outcome.

Recruitment organization

As this is a multicentre trial, different methods will be suitable and used by different participating study-sites in order to include patients. In general, patients will be informed about the study at their visit to the emergency department. Patients who are not subjected to further treatment in the hospital will either be included and randomized immediately after given both written and oral information about the study,

For peer review only - http://bmjopen.bmj.com/site/about/guidelines.xhtml

or if this is not possible, receive information about the study and scheduled for an outpatient study appointment within the following days. Patients who are in need of surgery and operated on within 10 days can be included and randomized, after receiving written and oral information. All participating patients will sign an informed consent form, which will be collected by the local study personnel and delivered to the central study organization for archiving and the end of the trial. The steering committee will aid recruiting centres in setting up the local infra-structure needed for patient recruitment.

Study Organization

11 hospitals from 7 different regions have so far accepted to participate in the study.

Study participants

1400 patients will be included; whereof approximately 1000 ankle fractures and 400 Achilles tendon ruptures.

- Inclusion criteria: age of 18-75 years, unilateral isolated ankle fracture or Achilles tendon rupture and able to be included in the study within 10 days of injury.

- Exclusion criteria: a patient that is unable to give informed consent, unable to follow instructions and unable to receive orthosis treatment, or has known kidney failure, heart failure with pitting oedema, malignancy, haemophilia, thrombophilia, current DVT or PE, pregnancy, or has follow-up planned at a hospital outside of the study organization.

For peer review only - http://bmjopen.bmj.com/site/about/guidelines.xhtml

Objective

The primary objective is to investigate if adjuvant IPC treatment during the duration of lower leg immobilization treatment will reduce the incidence of VTE in an outpatient setting.

Randomization

Patients will be randomly assigned to either standard treatment or standard treatment with adjuvant IPC with a 1:1 allocation, after receiving written and oral information and signed an informed consent form. The randomization is stratified by study site, patient over/under 40 years of age and by type of injury (AF or ATR) in blocks of 4. Randomization lists are uploaded to the REDCap online eCRF system (16) hosted at Karolinska Institutet, only accessible to the trials REDCap administrator. Randomization is only performed by instructed study personnel. All activities within REDCap are tracked, and the REDCap log is non-modifiable.

Intervention

The intervention, *addition of IPC to routine care*, will be administered in the emergency department, the outpatient clinic or the hospital ward. Patients randomized to the intervention will during the whole duration of lower limb immobilization receive calf IPC on each of the lower limbs (Venaflow® Elite, DJO LLC, Vista, CA, USA) applied under an orthosis (Aircast Airselect™ Elite Walker, DJO LLC, Vista, CA, USA). When used, the calf-cuffs will alternately inflate every 30 seconds. The patients will be instructed to apply the IPC-therapy as much as possible during the time they are sedentary, both day and night, at least 10 h/24 h. Patients should, however, not keep themselves from being active in order to comply with IPC-treatment. Compliance with the treatment will be registered by both the patient and by the device (2). IPC will be discontinued if the patient declines to

For peer review only - http://bmjopen.bmj.com/site/about/guidelines.xhtml

For peer review only

continue IPC or had adverse effects of the IPC that warrants removal. Patients that use the IPC for 30 hours/week or more will be deemed compliant to the treatment. IPC-treatment will be ended when the leg immobilization is removed.

Comparator

Patients randomized standard care will receive lower limb immobilization in an orthosis or a below-knee plaster cast according to local routines. The amount of weight-bearing allowed will be determined by the patient's physician. Weight-bearing will be recorded and accounted for in the statistical analysis.

Blinding

Blinding will not be possible for the patient or for the study personnel responsible for patient inclusion. Sonographers will be blinded to patient allocation.

Study outcomes

Primary Outcome measure

The primary outcome is VTE during the lower leg immobilized period. VTE will be assessed clinically if a patient develops symptoms of DVT or pulmonary embolism and verified by compression Duplex ultrasound or computer tomography, respectively. Moreover, DVT will be screened for by CDU at termination of leg immobilization.

Secondary outcome measure

Secondary outcome measures include:

- Patient Reported Outcome Measures (PROM) will be reported by all participating patients at 6- and 12 months after injury. The questionnaires EQ-5D-5L(17) and the Foot- and Ankle Outcome Score (FAOS)(18) will be

For peer review only - http://bmjopen.bmj.com/site/about/guidelines.xhtml

answered by all patients. In addition, the Olerud-Molander Outcome Score (OMAS)(19) will be answered by the ankle fracture patients and the Achilles tendon Total Rupture Score (ATRS)(20) by the Achilles tendon rupture patients. The data will be collected and managed using REDCap electronic data capture tool (16).

- Compliance to IPC-treatment. Patients randomized to IPC-treatment will give weekly reports regarding IPC-device usage. The IPC-device will also record the cumulative time when used.
- Post-injury weight-bearing. Weight-bearing will be recorded via patient recorded daily journals. The patient is asked to note how much weight they load on their injured foot while walking, on a scale from 0-100. 0 is equal to non-weight-bearing walking with the aid of crutches and 100 is fully weight-bearing in the orthosis/plaster cast on the injured foot without crutches. A recent study has shown that this scale analysis is a reliable method of assessing patient weight-bearing of the patient (21).
- Adverse events. Events such as post-operative infection, fracture dislocation, re-rupture or re-operation will be recorded. Furthermore, patient mortality will be registered at one year after injury.
- IPC-related adverse events. Events caused by IPC-treatment such as skin damage or infection, prematurely discontinuing IPC-treatment or fall injuries will be recorded.
- Microdialysis of Achilles tendon healing. Healing metabolites as well as callus production will be assessed at termination of leg immobilization, 6-8 weeks, using microdialysis in a subgroup of 50 ATR patients, followed by

For peer review only

For peer review only - http://bmjopen.bmj.com/site/about/guidelines.xhtml

quantification of procollagens, to assess tendon healing. Samples will be collected and stored at Stockholm medical biobank.

- Heel-rise endurance test. The heel-rise endurance test is an established functional test for patients with Achilles tendon ruptures and will be performed by a subgroup of patients at 1 year after injury, to measure muscle-tendon strength in the injured, compared to the uninjured leg. The MuscleLab (Ergo Test Technology, Stathelle, Norway) measurement system is used.
- VTE-preventive mechanisms. Assessment of profibrinolysis and coagulation factors as well as blood-flow quantification using ultrasound will be assessed in a subgroup of 50 patients. Overall hemostatic potential, D-dimer, Plasminogen activator inhibitor-1, endogen thrombin potential, trombin-antitrombin-complex, plasmin antiplasmin complex and tissue plasminogen activator/PAI complex will be assessed at Karolinska Institutet, Coagulation Unit. Samples will be collected and stored at Stockholm medical biobank.
- The incidence of VTE in patients using anticoagulants such as low molecular weight heparin (LMWH) with or without adjuvant IPC.
- Blood-flow quantification. Peak femoral blood velocity will be calculated using custom ultrasound software and compared with baseline values, while microcirculation and tissue oxygenation will be assessed by near-infrared spectroscopy (INVOS®). These explanatory continuous data will be compared between the groups.
- Cost-benefit analysis. Patient annual salary, type of occupation and the numbers of days on sick-leave will be recorded. This will then be used to calculate if the intervention is cost-effective in the included patients.

For peer review only - http://bmjopen.bmj.com/site/about/guidelines.xhtml

Time Schedule

Recruitment of patients began in August of 2018. Final inclusion is estimated to occur in August 2022.

Setup

Follow-up investigations

All patients will be followed as usual with an outpatient visit approximately at 2 weeks after injury/surgery where compliance and adverse events will be recorded. At the time of termination of treatment (usually 6-8 weeks after injury/surgery) the orthosis will be removed and the patient will be examined by an orthopaedic surgeon or physiotherapist, who will document compliance and adverse events. At this time the ultrasound investigation of the lower limbs will occur, following a standardized protocol. The patient will be treated according to local routines in the event of a venous thrombosis.

Patients will receive a journal where they will note the estimated amount of loading on their injured leg (0-100%) and, if randomized to intervention, how many hours they used IPC-treatment each day. This will be self-reported via the REDCap digital clinical report forms once a week and re-checked at the last out-patient when discontinuing the lower leg immobilization. Patients who do not have access to an e-mail address or do not want to report their data digitally, will hand in their journal at the discontinuation of immobilization, and the data will be entered manually by the research personnel.

Protocol for duplex ultrasound

Ultrasound will be performed throughout by trained sonographers. The investigation will be performed on the injured leg only. Locations investigated include the deep

For peer review only

veins (femoralis communis, femoralis superficialis, femoralis profunda, poplitea, tibialis posterior and fibularis) and muscular veins. A positive investigation is reported when any of the following is observed: visible thrombi in the 2D projection, a non-compressible vein, an expanded vein and lack of doppler signal or stripe flow. All positive findings will be validated by a sonographer or radiologist in the steering-committee.

Statistical analysis

Sample size

The STOP Leg Clots is designed on a hypothesis of superiority of adjuvant intermittent pneumatic compression therapy compared to standard treatment during lower leg immobilization in prevention of venous thromboembolism. Based on the existing literature the expected occurrence of DVT in the control group as detected by screening ultrasound is up to 25% for AF and 50% in ATR (2, 3). The estimates are dependent on the case-mix ratio as the incidence of DVT is higher in ATR than in AF. We expect the case-mix to be 30% ATR and 70% AF, resulting in an expected DVT-rate of 33%. As extra precautions we reduced the expected total DVT-rate from 33% to 28%. The expected relative risk reduction of DVT is 40% based on earlier studies (22), and we chose to calculate with a lower risk reduction value of 29%. This leads to an absolute risk reduction of DVT of 8%, which is higher than the suggested minimum clinically relevant absolute risk reduction of 4% (2, 22). The sample size calculations for the study are based on the following estimates and parameters: alpha = 0.05, 1-beta = 90%, estimated incidence of DVT = 28%, the smallest relative risk reduction to detect= 29%. The sample size needed using these study parameters is 597 in each group. Expecting a drop out rate of 5-10% and to compensate for the

For peer review only

uncertainty of the final case-mix distribution we aim to include 1400 patients in our study, around 1000 AF patients and 400 ATR patients.

Statistical analysis plan

Descriptive statistics

The flow of patients through the trial will be displayed according to the CONSORT statement. The number of patients screened; number of ineligible patients and the reasons why; number of eligible patients not providing consent; and the number of eligible patients subsequently randomised will be presented. The characteristics of the screened population, the ineligible participants and eligible participants who consent and do not consent will be summarised. Information regarding the number of patients that are surgically treated versus those who are treated non-operatively will be presented. Data on patient eligibility and reasons for withdrawal from treatment or the trial (when stated) will be summarised. Baseline patient characteristics will be summarized using descriptive statistics; counts for categorical variables and mean/median and inter-quartile range for continuous variables.

General data handling

All analyses will be conducted blinded for the treatment allocation and with pseudonymized data. All statistical tests will be two-sided with an α of 0.05. Primary analyses will be by intention-to-treat. However, per-protocol and as-treated analyses will also be performed. The final pseudonymized data-set will be stored separate from the pseudonymization key on secure hospital and/or university servers, in order to preserve patient confidentiality. Any paper forms completed by the patients are delivered at the end of the trial to the central study organisation.

For peer review only - http://bmjopen.bmj.com/site/about/guidelines.xhtml

Data analyses

Primary outcome

The primary outcome will be presented as odds ratios and 95% confidence intervals, adjusted using logistic regression for the factors weight-bearing, anticoagulant treatment, time to treatment, surgery vs. no surgery and patient characteristics. Absolute reductions in risk of VTE will be calculated from these values, as presented in the reference with similar study design (22).

Secondary outcome

Group differences for ordinal secondary data, such as the patient-reported data, will be analysed by nonparametric tests. Group differences for index scale, ratio scale and continuous secondary outcome will be analysed with appropriate parametric or non-parametric tests.

Missing data

Missing data will be investigated and analysed. Reasons for missing data will be stated and summarized where possible. Sensitivity analyses will be performed in order to test the strength and consistency of the final results.

Monitoring/auditing

Research nurses educated in monitoring research studies will perform study monitoring during the trial. Auditing of the trial will be performed by Karolinska Trial Alliance, which is an independent organization from the investigators and sponsor.

Safety

Risk and side effect

All included patients have an increased risk of developing DVT and PE. There will be no general recommendation for included patients for pharmacoprophylaxis, in line with international guidelines (23, 24). The steering committee advice only to prescribe pharmacoprophylactic drugs to patients with other identifiable risk factors such as

For peer review only

previous DVT/PE and usage of birth-control pills. The use of pharmacoprophylaxis will, however, not be an exclusion criterion. Patients with prescription of anticoagulants will be adjusted for in the statistical analysis.

Adverse events and complications

Adverse events in connection with the usage of the intervention (IPC and orthosis) or casting will be noted when reported in the study protocol and referred to the responsible physician if needed. Complications such as infection, fracture dislocation, tendon re-rupture, reoperation or serious life-threatening adverse events will be captured within the clinical injury treatment protocol of the local hospital. Minor or major complications may also be noted through the detailed study protocol and referred to the responsible physician if needed.

Discussion

Overall aim of the trial

There is an urgent need for more efficient and safer VTE-prophylactics, especially for lower limb immobilized patients, according to major health service authorities (23, 24). The overall aim of STOP Leg Clots is to investigate whether or not adjuvant intermittent pneumatic compression treatment is reliably effective in outpatient lower leg immobilized patient. We therefore chose two types of injuries, Achilles tendon ruptures and ankle fractures, which constitute a large portion of patients with lower leg immobilization that also have a significantly increased risk of VTE (25). Moreover, patients with ATR and AF have demonstrated low- or non-effectiveness of LMHW prophylaxis also in relation to the risk of bleeding complications (6, 8, 25). In the perspective that different hospitals have different clinical traditions and indications for surgery of ATR and AF we have chosen to include both operated and non-operated ATR/AF, since both groups are equally served by VTE prophylaxis. Earlier studies

For peer review only - http://bmjopen.bmj.com/site/about/guidelines.xhtml

have shown a just as high incidence of VTE in non-operated as in operated ATR/AF (26), further indicating a benefit of demonstrating a VTE risk reduction in both operated/non-operated lower limb immobilized patients.

Compliance

Compliance to the allocated treatment, specifically number of days and hours of IPC usage, is key in understanding if and how the intervention provide an effect on the primary outcome. This will be monitored by patient self-assessment diaries together with the built-in timer in the IPC-device. American College of Chest Physicians recommend that efforts should be made to achieve 18 h of daily compliance with IPC in hospitalized patients (27). However, there are no recommendations for mobile outpatients. In this study we will recommend at least 10h of bilateral IPC-treatment per 24 hours. However, patients should not be more sedentary than otherwise in order to comply with treatment. Patients will be considered compliant with the intervention if used for 30 hours/week throughout the duration of lower limb immobilization.

Endpoints

Compression doppler ultrasonography is the investigation of choice to confirm the diagnosis of DVT since it is non-invasive, acceptable to patients, easily repeated several times and uses equipment widely available (28). Thus consent, accrual and adherence in a trial would more likely be achieved than with alternatives such as phlebography.

For peer review only - http://bmjopen.bmj.com/site/about/guidelines.xhtml

Ethics and dissemination of results

Ethical approval was obtained by the ERB in Stockholm, Sweden, Dnr 2016/1573-31. The results of this study will provide evidence-based treatment protocol for leg immobilized patients. The study results will be submitted for publication to international, peer-reviewed journals, regardless of whether the results are positive, negative or inconclusive in relation to the study hypothesis.

Availability of data and material

The final anonymized data will be available for the PI and the steering-committee and also made available upon a reasonable request.

Patient and public involvement

A patient representative is on the steering committee of the research project to help in planning and improving the relevance, quality and validity of the research. The patient representative will also receive appropriate training for the job and will actively participate in the development of recommendations and guidelines.

Competing interests

None of the investigators have any financial or other competing interest in relation to the trial. DJO Global is the manufacturer of the IPC-devices used in the study and have made them available for study sites to rent throughout the study. Disposable materials (cuffs and connective tubing) are bought from the DJO Gloabal, Inc. DJO Global has no influence in the conduct of the trial or publication of the results.

Funding

The study is financed by the Swedish research council (Dnr: 2017-00202).

Authors' contributions

All authors have contributed to the design of the present trial protocol. PWA is the primary investigator and initiated the study. BA, HB, EDA, KJ and KNH are all helping with study implementation.The protocol was drafted by PWA, SS and refined by BA,

For peer review only - http://bmjopen.bmj.com/site/about/guidelines.xhtml

HB, EDA, KJ and KNH. SS was responsible for drafting of the manuscript. All authors have read and approved the final manuscript.

Acknowledgements

The STOP Leg Clots trial is currently including patients from the following centers: Akademiska Universitetssjukhuset (Kenneth Jonsson, Catharina Strömstedt), Centralsjukhuset Karlstad (Markus Lundmark, Pendar Khalili), Danderyd Hospital (Erica Domeij-Arverud, Sophia Grindberg, Paula-Therese Kelly, Marie Ax), Gävle Sjukhus (Irina Kolioumpakina Akrioti, Hans Peter Bögl), Helsingborgs lasarett (Anna-Karin Svensson), Höglandssjukhuset (Björn Alkner), Karolinska Universitetssjukhuset (Paul Ackermann, Susanna Aufwerber, Luigi Belcastro, Hans Berg, Johanna Flodin, Malin Heijkenskjöld, Simon Svedman, Maria Wiksén), Norrtälje Sjukhus (Per-Anton Svensson), Sahlgrenska Universitetssjukhuset (Katarina Nilsson-Helander, Niklas Nilsson), Södersjukhuset (Lasse Lapidus, Elisabeth-Skogman, Annika Tiliander), Östersunds sjukhus (Harry Mitchell, Alexander De Val Olsson, Jörgen Larsson).

References

1. Mammen EF. Pathogenesis of venous thrombosis. Chest. 1992;102(6 Suppl):640S-4S.
2. Domeij-Arverud E, Labruto F, Latifi A, Nilsson G, Edman G, Ackermann PW. Intermittent pneumatic compression reduces the risk of deep vein thrombosis during post-operative lower limb immobilisation - a prospective randomised trial of acute ruptures of the Achilles tendon. Bone Joint J 2015;97(B(5)):675-80.
3. Lapidus LJ, Ponzer S, Elvin A, Levander C, Larfars G, Rosfors S, et al. Prolonged thromboprophylaxis with Dalteparin during immobilization after ankle fracture surgery: a randomized placebo-controlled, double-blind study. Acta Orthop. 2007;78(4):528-35.
4. Zee AA, van Lieshout K, van der Heide M, Janssen L, Janzing HM. Low molecular weight heparin for prevention of venous thromboembolism in patients with lower-limb immobilization. Cochrane Database Syst Rev. 2017;8:CD006681.
5. Jorgensen PS, Warming T, Hansen K, Paltved C, Vibeke Berg H, Jensen R, et al. Low molecular weight heparin (Innohep) as thromboprophylaxis in outpatients with a plaster cast: a venografic controlled study. Thrombosis research. 2002;105(6):477-80.
6. Lapidus LJ, Rosfors S, Ponzer S, Levander C, Elvin A, Larfars G, et al. Prolonged thromboprophylaxis with dalteparin after surgical treatment of achilles tendon rupture: a randomized, placebo-controlled study. J Orthop Trauma. 2007;21(1):52-7.
7. van Adrichem RA, van Oosten JP, Cannegieter SC, Schipper IB, Nelissen RG. Thromboprophylaxis for lower leg cast immobilisation and knee arthroscopy: a survey study. Neth J Med. 2015;73(1):23-9.

For peer review only

8. Falck-Ytter Y, Francis CW, Johanson NA, Curley C, Dahl OE, Schulman S, et al. Prevention of VTE in orthopedic surgery patients: Antithrombotic Therapy and Prevention of Thrombosis, 9th ed: American College of Chest Physicians Evidence-Based Clinical Practice Guidelines. Chest. 2012;141(2 Suppl):e278S-325S.
9. Street JT, McGrath M, O'Regan K, Wakai A, McGuinness A, Redmond HP. Thromboprophylaxis using a low molecular weight heparin delays fracture repair. Clin Orthop Relat Res. 2000(381):278-89.
10. Treasure T. Venous thromboembolism: reducing the risk In: Excellence NIfHaC, editor. CG92 ed. London: National Institute for Health and Clinical Excellence; 2010. p. 24-8.
11. Cohen AT, Agnelli G, Anderson FA, Arcelus JI, Bergqvist D, Brecht JG, et al. Venous thromboembolism (VTE) in Europe. The number of VTE events and associated morbidity and mortality. Thromb Haemost. 2007;98(4):756-64.
12. Statens beredning för medicinsk och social utvärdering. Blodpropp - förebyggande, diagnostik och behandling av venös tromboembolism en systematisk kunskapssammanställning / Vol. 1. Stockholm: SBU,; 2002. Available from: http://www.sbu.se/upload/Publikationer/Content0/1/blodpropp_2002/blodproppfull.html.
13. Barrera LM, Perel P, Ker K, Cirocchi R, Farinella E, Morales Uribe CH. Thromboprophylaxis for trauma patients. Cochrane Database Syst Rev. 2013(3):CD008303.
14. Alim MA, Svedman S, Edman G, Ackermann PW. Procollagen markers in microdialysate can predict patient outcome after Achilles tendon rupture. BMJ Open Sport & Exercise Medicine. 2016;2(1).
15. Chan AW, Tetzlaff JM, Altman DG, Laupacis A, Gotzsche PC, Krleza-Jeric K, et al. SPIRIT 2013 statement: defining standard protocol items for clinical trials. Ann Intern Med. 2013;158(3):200-7.
16. Harris PA, Taylor R, Thielke R, Payne J, Gonzalez N, Conde JG. Research electronic data capture (REDCap)--a metadata-driven methodology and workflow process for providing translational research informatics support. J Biomed Inform. 2009;42(2):377-81.
17. Devlin NJ, Shah KK, Feng Y, Mulhern B, van Hout B. Valuing health-related quality of life: An EQ-5D-5L value set for England. Health Econ. 2018;27(1):7-22.
18. Roos EM, Brandsson S, Karlsson J. Validation of the foot and ankle outcome score for ankle ligament reconstruction. Foot Ankle Int. 2001;22(10):788-94.
19. Nilsson GM, Eneroth M, Ekdahl CS. The Swedish version of OMAS is a reliable and valid outcome measure for patients with ankle fractures. BMC Musculoskelet Disord. 2013;14:109.
20. Nilsson-Helander K, Thomee R, Silbernagel KG, Thomee P, Faxen E, Eriksson BI, et al. The Achilles tendon Total Rupture Score (ATRS): development and validation. Am J Sports Med. 2007;35(3):421-6.
21. Aufwerber S, Heijne A, Gravare Silbernagel K, Ackermann PW. High Plantar Force Loading After Achilles Tendon Rupture Repair With Early Functional Mobilization. Am J Sports Med. 2019;47(4):894-900.
22. Collaboration CT, Dennis M, Sandercock P, Reid J, Graham C, Forbes J, et al. Effectiveness of intermittent pneumatic compression in reduction of risk of deep vein thrombosis in patients who have had a stroke (CLOTS 3): a multicentre randomised controlled trial. Lancet. 2013;382(9891):516-24.
23. Falck-Ytter Y, Francis CW, Johanson NA, Curley C, Dahl OE, Schulman S, et al. Prevention of VTE in orthopedic surgery patients: Antithrombotic Therapy and Prevention of Thrombosis, 9th ed: American College of Chest Physicians Evidence-Based Clinical Practice Guidelines. Chest. 2012;141(2 Suppl):e278S-e325S.
24. Treasure T, Hill J. NICE guidance on reducing the risk of venous thromboembolism in patients admitted to hospital. J R Soc Med. 2010;103(6):210-2.
25. Calder JD, Freeman R, Domeij-Arverud E, van Dijk CN, Ackermann PW. Meta-analysis and suggested guidelines for prevention of venous thromboembolism (VTE) in foot and ankle surgery. Knee Surg Sports Traumatol Arthrosc. 2016;24(4):1409-20.

For peer review only - http://bmjopen.bmj.com/site/about/guidelines.xhtml

26. Batra S, Kurup H, Gul A, Andrew JG. Thromboprophylaxis following cast immobilisation for lower limb injuries--survey of current practice in United Kingdom. Injury. 2006;37(9):813-7.
27. Fox G, Gabbe BJ, Richardson M, Oppy A, Page R, Edwards ER, et al. Twelve-month outcomes following surgical repair of the Achilles tendon. Injury. 2016;47(10):2370-4.
28. Sampson FC, Goodacre S, Kelly AM, Kerr D. How is deep vein thrombosis diagnosed and managed in UK and Australian emergency departments? Emerg Med J. 2005;22(11):780-2.

For peer review only

For peer review only - http://bmjopen.bmj.com/site/about/guidelines.xhtml

BMJ Open

STOP Leg Clots - Swedish multicenter Trial of Outpatient Prevention of Leg Clots: study protocol for a randomized controlled trial on the efficacy of intermittent pneumatic compression on venous thromboembolism in lower leg immobilized patients

Journal:	*BMJ Open*
Manuscript ID	bmjopen-2020-044103.R1
Article Type:	Protocol
Date Submitted by the Author:	01-Mar-2021

Complete List of Authors:	Svedman, Simon; Karolinska Institutet, Molecular Medicine and Surgery; Karolinska University Hospital, Department of Orthopedic Surgery<br>Alkner, Björn; Linköping University, Department of Orthopaedics, Eksjö, Region Jönköping County and Department of Biomedical Clinical Science<br>Berg, Hans; Karolinska University Hospital, Department of Orthopedic Surgery; Karolinska institutet Department of Clinical Sciences Intervention and Technology, Division of Orthopaedics and Biotechnology<br>Domeij-Arverud, Erica; Karolinska Institutet, Department och clinical sciences, Danderyd Hospital<br>Jonsson, Kenneth; Uppsala University Hospital, Department of Surgical Sciences<br>Nilsson Helander, Katarina; Sahlgrenska University Hospital, Department of Orhopaedics<br>Ackermann, Paul; Karolinska Institutet, Department of Molecular Medicine and Surgery; Karolinska University Hospital, Department of Orthopedic Surgery

**Primary Subject Heading**:	Sports and exercise medicine
Secondary Subject Heading:	Haematology (incl blood transfusion)
Keywords:	Foot & ankle < ORTHOPAEDIC & TRAUMA SURGERY, Thromboembolism < CARDIOLOGY, Anticoagulation < HAEMATOLOGY, Orthopaedic sports trauma < ORTHOPAEDIC & TRAUMA SURGERY, Ultrasound < RADIOLOGY & IMAGING, Orthopaedic & trauma surgery < SURGERY

SCHOLARONE™
Manuscripts

I, the Submitting Author has the right to grant and does grant on behalf of all authors of the Work (as defined in the below author licence), an exclusive licence and/or a non-exclusive licence for contributions from authors who are: i) UK Crown employees; ii) where BMJ has agreed a CC-BY licence shall apply, and/or iii) in accordance with the terms applicable for US Federal Government officers or employees acting as part of their official duties; on a worldwide, perpetual, irrevocable, royalty-free basis to BMJ Publishing Group Ltd ("BMJ") its licensees and where the relevant Journal is co-owned by BMJ to the co-owners of the Journal, to publish the Work in this journal and any other BMJ products and to exploit all rights, as set out in our licence.

The Submitting Author accepts and understands that any supply made under these terms is made by BMJ to the Submitting Author unless you are acting as an employee on behalf of your employer or a postgraduate student of an affiliated institution which is paying any applicable article publishing charge ("APC") for Open Access articles. Where the Submitting Author wishes to make the Work available on an Open Access basis (and intends to pay the relevant APC), the terms of reuse of such Open Access shall be governed by a Creative Commons licence – details of these licences and which Creative Commons licence will apply to this Work are set out in our licence referred to above.

Other than as permitted in any relevant BMJ Author's Self Archiving Policies, I confirm this Work has not been accepted for publication elsewhere, is not being considered for publication elsewhere and does not duplicate material already published. I confirm all authors consent to publication of this Work and authorise the granting of this licence.

For peer review only

STOP Leg Clots - Swedish multicenter Trial of Outpatient Prevention of Leg Clots: study protocol for a randomized controlled trial on the efficacy of intermittent pneumatic compression on venous thromboembolism in lower leg immobilized patients

Simon Svedman[1,2], Björn A. Alkner[3], Hans E Berg[2,4], Erica Domeij-Arverud[5], Kenneth B. Jonsson[6], Katarina Nilsson Helander[7], Paul W. Ackermann[1,2]

1 - Department of Molecular Medicine and Surgery, Karolinska Institutet, Stockholm, Sweden.

2 - Department of Orthopaedic Surgery, Karolinska University Hospital, Stockholm, Sweden

3 - Department of Orthopaedics, Eksjö, Region Jönköping County and Department of Biomedical Clinical Science, Linköping University, Linköping, Sweden

4 - Division of Orthopaedics and Biotechnology, Department of Clinical Science, Intervention and Technology, Karolinska Institutet, Stockholm, Sweden

5 - Karolinska Institutet, Department of Clinical Sciences, Danderyd Hospital, Stockholm, Sweden.

6 - Department of Surgical Sciences, Uppsala University Hospital, Uppsala, Sweden

7 - Department of Orthopaedics, Sahlgrenska University Hospital, Gothenburg, Sweden

Email

Simon.svedman@ki.se, bjorn.alkner@liu.se, hans.er.berg@sll.se, Erica.domeij-arverud@sll.se, kenneth.jonsson@surgsci.uu.se, katarina.nilsson.helander@vgregion.se, Paul.ackermann@ki.se

Corresponding author:

Paul W. Ackermann;
Karolinska Universitetssjukhuset, Ortopedmottagningen A6:01, 171 76 Stockholm
Fax: +46851772695, Phone: +46851771734, Email: Paul.ackermann@ki.se

For peer review only

Abstract:

Introduction:

Leg immobilization in a cast or an orthosis after lower limb injuries is associated with a high risk of complications of venous thromboembolism (VTE) and hampered healing. Current pharmacoprophylaxes of VTE are inefficient and associated with adverse events. Intermittent pneumatic compression (IPC) could represent a novel, efficient and safe VTE-prophylactic alternative that may enhance injury healing. The aim of STOP leg clots is to demonstrate that adjuvant IPC-therapy reduces VTE-incidence and improves healing in lower leg immobilized outpatients.

Methods and analysis:

STOP leg clots is a multicentre randomised controlled superiority trial. Eligible patients (700 patients/arm) with either an acute ankle fracture or Achilles tendon rupture will be randomized to either addition of IPC during lower-leg immobilization or to treatment-as-usual. The primary outcome will be the total VTE-incidence, i.e. symptomatic and asymptomatic deep venous thrombosis (DVT) or symptomatic pulmonary embolism (PE), during the leg immobilization period, approximately 6-8 weeks. DVT-incidence will be assessed by screening whole leg compression duplex ultrasound at removal of leg immobilization and/or clinically diagnosed within the time of immobilization. Symptomatic PE will be verified by computer tomography.

Secondary outcomes will include patient-reported outcome using validated questionnaires, healing evaluated by measurements of tendon callus production and changes in VTE-prophylactic mechanisms assessed by blood flow and fibrinolysis. Data analyses will be blinded and based on the intention-to-treat.

For peer review only - http://bmjopen.bmj.com/site/about/guidelines.xhtml

Ethics and dissemination:

Ethical approval was obtained by the ethical review board in Stockholm, Sweden, Dnr 2016/1573-31. The study will be conducted in accordance with the Helsinki declaration. The results of the study will be disseminated in peer-reviewed international journals.

Trial Registration:

ClinicalTrials.gov Identifier: NCT03259204, registered August 23, 2017.

Strengths and limitations of this study

- This is the first multicentre randomized controlled trial to investigate the efficiency of adjuvant IPC on VTE for the duration of ankle joint immobilization in an outpatient setting.
- The multicenter design improves the external validity of the study and will allow rapid dissemination and potential implementation of study results.
- Patients cannot be blinded to allocation.
- Clinical sonographers are responsible to assess the primary outcome and will be blinded to patient allocation.
- The duplex ultrasound screening will detect subclinical VTE which will allow high study power. However, the clinical relevance of non-symptomatic events is still not fully clear.

For peer review only

For peer review only - http://bmjopen.bmj.com/site/about/guidelines.xhtml

Introduction

Venous thromboembolism (VTE), comprising deep venous thrombosis (DVT) and pulmonary embolism (PE), is a critical medical condition and potentially deadly. An estimated 370.000 people die of VTE in Europe each year, and more than 700.000 Europeans are afflicted(1). Patients who survive often suffer chronic ailments with significantly lower quality of life after an event (2-7). Leg immobilization in a cast or orthosis is a common step in the treatment of patients with lower limb injury, and commonly causes VTE due to venous stasis (8). Estimated DVT incidence may range from 21% up to 50% after ankle fractures (AF) and Achilles tendon ruptures (ATR), respectively (9, 10).

For peer review only

The most widely used method to prevent VTE during lower leg immobilization is through pharmacological prophylaxis, such as low molecular weight heparin (LMWH). However, several studies have demonstrated only a marginal or no effect of LMWH in lower leg immobilized patients and is not cost efficient (7, 11-15). In addition to their low efficacy, LMWH increases the risk of bleeding complications (13, 14) and may impair tissue healing (16). For these reasons, the National Health Service in UK, and the corresponding authority in Sweden, have stressed the need for safer and more efficient VTE-prophylaxis (17-19).

Mechanical thromboprophylaxis with intermittent pneumatic compression (IPC) reduces VTE, is a cost-effective intervention in hospitalized patients, and is recommended by guidelines for surgical in-hospital patients (17, 20). Whether long-term IPC treatment applied during leg immobilization in an outpatient setting can reduce the incidence of DVT is unknown. We recently published a study presenting preliminary evidence that leg immobilized patients with ATR treated with adjuvant

For peer review only - http://bmjopen.bmj.com/site/about/guidelines.xhtml

IPC at 2 weeks had a reduced DVT rate (21% vs. 37%) compared to controls using Doppler ultrasound screening. However, in the previous study the IPC intervention ended at two weeks and at 6 weeks the DVT-rate was 50% in both groups (9).

Thus, the primary aim of this prospective, multicentre, randomized controlled trial is to provide reliable evidence of the efficacy of prolonged adjuvant IPC therapy in leg-immobilized patients on the total incidence of VTE up to removal of leg immobilization.

Lower leg immobilization, in addition to increased risk of VTE, may also lead to impaired healing after initial injury as well as long lasting muscle atrophy (21). As recent studies suggest that the increased neuro-vascular flow induced by IPC could trigger the healing process (22), we also aim to assess whether adjuvant IPC enhances healing, reduced muscle atrophy and improves the long-term patient-reported outcomes in lower limb immobilized patients.

Methods and analysis

This is a report of the trial protocol dated 4th September 2017, supported by the Swedish research council. Any important protocol modifications will be communicated through ClinicalTrials.gov trial number NCT03259204, the Ethical Review Board and/or the BMJ Open journal. The protocol was developed in accordance with Standard Protocol Items: Recommendations for Interventional Trials and Template for Intervention Description and Replication statements (23).

Study design and setting

“STOP leg clots” is a multicentre, prospective, superiority, randomized, controlled, parallel group trial with blinded assessment of the outcome.

For peer review only - http://bmjopen.bmj.com/site/about/guidelines.xhtml

Recruitment organization

In general, patients will be informed about the study at their visit to the emergency department. As this is a multicentre trial, different methods will be suitable and used by different participating study-sites in order to include patients. Patients who are not subjected to further treatment in the hospital will either be included and randomized immediately after given both written and oral information about the study, or if this is not possible, receive information about the study and scheduled for an outpatient study appointment within the following days. Patients who are in need of surgery and operated on within 10 days can be included and randomized, after receiving written and oral information. All participating patients will sign an informed consent form, which will be collected by the local study personnel and delivered to the central study organization for archiving and the end of the trial. The steering committee will aid recruiting centres in setting up the local infra-structure needed for patient recruitment.

Study Organization

11 hospitals from 7 different Swedish regions and 2 hospitals from 2 other European countries have so far accepted to participate in the study.

Study participants

1400 patients will be included; whereof approximately 1000 ankle fractures and 400 Achilles tendon ruptures.

- Inclusion criteria: age of 18-75 years, unilateral isolated ankle fracture or Achilles tendon rupture and able to be included in the study within 10 days of injury.

- Exclusion criteria: unable to give informed consent, unable to follow instructions and unable to receive orthosis treatment, known kidney failure, heart failure with pitting oedema, malignancy, haemophilia, thrombophilia,

For peer review only - http://bmjopen.bmj.com/site/about/guidelines.xhtml

current DVT or PE, pregnancy, or has follow-up planned at a hospital outside of the study organization.

Objective

The primary objective is to investigate if adjuvant IPC treatment during the duration of lower leg immobilization treatment will reduce the incidence of VTE in an outpatient setting.

Randomization

Patients will prior to randomization receive both written and oral information and sign an informed consent form. Patients will be randomly assigned to either standard treatment or standard treatment with adjuvant IPC in an orthosis with a 1:1 allocation. Patient randomization may occur up to 10 days after the injury. The number of days from injury to start of randomized treatment as well as potential immobilization will be noted. The randomization is stratified by study site, patient age over/under 40 years and by type of injury (AF or ATR). Randomization occurs in blocks of 4. Randomization lists are uploaded to the REDCap online eCRF system (24) hosted at Karolinska Institutet, only accessible to the trials REDCap administrator. Randomization is only performed by instructed study personnel. All activities within REDCap are tracked, and the REDCap log is non-modifiable.

Intervention

The intervention, *addition of IPC to routine care*, will be administered in the emergency department, the outpatient clinic or the hospital ward. Patients randomized to the intervention will during the whole duration of lower limb immobilization receive bilateral calf-IPC (Venaflow® Elite, DJO LLC, Vista, CA, USA), on the injured side applied under an orthosis (Aircast Airselect™ Elite Walker, DJO LLC, Vista, CA, USA). Calf-IPC has previously been successfully used under an orthosis (9). Patients wearing an orthosis are instructed to open the orthosis 2-3

For peer review only - http://bmjopen.bmj.com/site/about/guidelines.xhtml

times per day while sedentary to reduce the humidity in the orthosis and prevent blistering. When IPC is used, the calf-cuffs will alternately inflate every 30 seconds. The patients will be instructed to apply the IPC-therapy as much as possible during the time they are sedentary, both day and night, with a goal of 10 h/24 h. Patients should, however, not keep themselves from being active in order to comply with IPC-treatment. Compliance with the treatment will be registered by both the patient and by the device (9). IPC will be discontinued if the patient declines to continue IPC or had adverse effects of the IPC that warrants removal. Patients that use the IPC for 30 hours/week or more will be deemed compliant to the treatment. IPC-treatment will be ended when the leg immobilization is removed.

Comparator

Patients randomized to standard care will receive lower limb immobilization in an orthosis or a below-knee plaster cast according to local routines. There has previously not been shown any difference in the incidence of VTE between plaster cast immobilization and orthosis-treatment (25). As standard care varies according to local routines and type of injury regarding duration of immobilization as well as weight-bearing these factors will be recorded and analysed for.

Intake of pharmacological antithrombotic drugs before the injury or administered during the immobilization period will be registered for category, dosage and duration. There is no general recommendation for the prescription of prophylactic pharmacological drugs in the trial for patients without any previously known risk factors for thrombosis.

For peer review only

For peer review only - http://bmjopen.bmj.com/site/about/guidelines.xhtml

Study outcomes

Primary Outcome measure

The primary outcome is VTE during the lower leg immobilized period. VTE will be assessed clinically if a patient develops symptoms of DVT or PE and verified by compression Duplex ultrasound or computer tomography, respectively. Moreover, DVT will be screened for by whole leg CDU of the injured leg at termination of leg immobilization by an experienced sonographer, which is 6-8 weeks after injury, depending on the type of injury and treatment used.

Secondary outcome measure

Secondary outcome planned for the study are listed below. Many of the secondary outcome measures need a much smaller number of patients included and will therefore only be performed on a limited number of sites to decrease the risk of methodological errors.

Secondary outcome measures include:

- Patient Reported Outcome Measures (PROM) will be reported by all participating patients at 6- and 12 months after injury. The questionnaires EQ-5D-5L (26) and the Foot- and Ankle Outcome Score (FAOS) (27) will be answered by all patients. In addition, the Olerud-Molander Outcome Score (OMAS) (28) will be answered by the ankle fracture patients and the Achilles tendon Total Rupture Score (ATRS) (29) by the Achilles tendon rupture patients. The data will be collected and managed using REDCap electronic data capture tool (24).
- Compliance to IPC-treatment. Patients randomized to IPC-treatment will give weekly reports regarding IPC-device usage by daily journals. The IPC-device will also record the cumulative time when used.

For peer review only - http://bmjopen.bmj.com/site/about/guidelines.xhtml

- Post-injury weight-bearing. Weight-bearing after injury have been suggested to improve lower limb-blood flow and decrease risk of DVT (25). Weight-bearing will be recorded via patient recorded daily journals. The patient is asked to note how much weight they load on their injured foot while walking, on a scale from 0-100. 0 is equal to non-weight-bearing walking with the aid of crutches and 100 is fully weight-bearing in the orthosis/plaster cast on the injured foot without crutches. A recent study has shown that this scale analysis is a reliable method of assessing weight-bearing of the patient (30).
- Adverse events. Events such as post-operative infection, fracture dislocation, re-rupture or re-operation will be recorded. Furthermore, patient mortality will be registered at one year after injury.
- IPC-related adverse events. Events caused by IPC-treatment such as skin damage or infection, prematurely discontinuing IPC-treatment or fall injuries will be recorded.
- Microdialysis of Achilles tendon healing. Healing metabolites as well as callus production will be assessed at termination of leg immobilization, 6-8 weeks, using microdialysis in a subgroup of 50 ATR patients, followed by quantification of procollagens, to assess tendon healing. Microdialysis is a well-known process for this aim, where a small catheter is inserted in the paratenon space (31). Samples will be collected and stored at Stockholm medical biobank.
- Heel-rise endurance test. The heel-rise endurance test is an established functional test for patients with Achilles tendon ruptures and will be performed by a subgroup of patients at 1 year after injury, to measure muscle-tendon

For peer review only - http://bmjopen.bmj.com/site/about/guidelines.xhtml

strength in the injured, compared to the uninjured leg. The MuscleLab (Ergo Test Technology, Stathelle, Norway) measurement system is used.

- VTE-preventive mechanisms. Assessment of fibrinolysis and coagulation factors as well as blood-flow quantification using ultrasound will be assessed in a subgroup of 50 patients. Overall hemostatic potential, D-dimer, Plasminogen activator inhibitor-1, endogen thrombin potential, trombin-antitrombin-complex, plasmin antiplasmin complex and tissue plasminogen activator/PAI complex will be assessed at Karolinska Institutet, Coagulation Unit. Samples will be collected from patients willing to participate at discontinuation of lower limb immobilization, prepared and stored at Stockholm medical biobank until analysis.
- The incidence of VTE in patients using anticoagulants such as low molecular weight heparin (LMWH) with or without adjuvant IPC will be analysed.
- Blood-flow quantification. Peak popliteal and femoral blood velocity will be calculated using custom ultrasound software and compared with baseline values, while microcirculation and tissue oxygenation will be assessed by near-infrared spectroscopy (INVOS®). These explanatory continuous data will be compared between the groups.
- Cost-benefit analysis. Patient annual salary, type of occupation and the numbers of days on sick-leave will be recorded. These data will be used in calculating cost-effectiveness for the prevention of VTE.

Blinding

Blinding will not be possible for the patient or for the study personnel responsible for patient inclusion. Sonographers will be blinded to patient allocation since the immobilization will be removed prior to ultrasound examination. All patients are

For peer review only - http://bmjopen.bmj.com/site/about/guidelines.xhtml

instructed not to disclose randomization allocation to the sonographers, and the sonographers are informed not to ask about this information.

Time Schedule

Recruitment of patients began in August of 2018. Final inclusion is estimated to occur in December 2022.

Setup

Follow-up investigations

All patients will be followed as usual with an outpatient visit approximately at 2 weeks after injury/surgery where compliance and adverse events will be recorded. At the time of termination of treatment (usually 6-8 weeks after injury/surgery) the orthosis will be removed and the patient will be examined by an orthopaedic surgeon or a physiotherapist, who will document compliance and adverse events. At this time the ultrasound investigation of the lower limbs will occur, following a standardized protocol. The patient will be treated according to local routines in the event of a venous thrombosis.

Patients will receive a journal where they will note the estimated amount of loading on their injured leg (0-100%) and, if randomized to intervention, how many hours they used IPC-treatment each day. This will be self-reported via the REDCap digital clinical report forms once a week and re-checked at the last out-patient when discontinuing the lower leg immobilization. Patients who do not have access to an e-mail address or do not want to report their data digitally, will hand in their journal at the discontinuation of immobilization, and the data will be entered manually by the research personnel.

Protocol for duplex ultrasound

Ultrasound will be performed throughout by trained sonographers on the injured leg only. Locations investigated include the deep veins (femoralis communis, femoralis

For peer review only - http://bmjopen.bmj.com/site/about/guidelines.xhtml

superficialis, femoralis profunda, poplitea, tibialis posterior and fibularis) and muscular veins. A positive investigation is reported when any of the following is observed: visible thrombi in the 2D projection, a non-compressible vein, an expanded vein and lack of doppler signal or stripe flow. All positive findings will be validated by a sonographer or radiologist in the steering-committee.

Statistical analysis

Sample size

The STOP Leg Clots is designed on a hypothesis of superiority of adjuvant intermittent pneumatic compression therapy compared to standard treatment during lower leg immobilization in prevention of venous thromboembolism. Based on the existing literature the expected occurrence of DVT in the control group as detected by screening ultrasound is up to 25% for AF and 50% in ATR (9, 10). The estimates are dependent on the case-mix ratio as the incidence of DVT is higher in ATR than in AF. We expect the case-mix to be 30% ATR and 70% AF, resulting in an expected DVT-rate of 33%. As extra precautions we reduced the expected total DVT-rate from 33% to 28%. The expected relative risk reduction of DVT is 40% based on earlier studies (32), and we chose to calculate with a lower risk reduction value of 29%. This leads to an absolute risk reduction of DVT of 8%, which is higher than the suggested minimum clinically relevant absolute risk reduction of 4% (9, 32). The sample size calculations for the study are based on the following estimates and parameters: alpha = 0.05, 1-beta = 90%, estimated incidence of DVT = 28%, the smallest relative risk reduction to detect= 29%. The sample size needed using these study parameters is 597 in each group. Expecting a drop-out rate of 5-10% and to compensate for the uncertainty of the final case-mix distribution we aim to include 1400 patients in our study, around 1000 AF patients and 400 ATR patients.

For peer review only - http://bmjopen.bmj.com/site/about/guidelines.xhtml

Statistical analysis plan

Descriptive statistics

The flow of patients through the trial will be displayed according to the CONSORT statement. The number of patients screened; number of ineligible patients and the reasons why; number of eligible patients not providing consent; and the number of eligible patients subsequently randomised will be presented. The characteristics of the screened population, the ineligible participants and eligible participants who consent and do not consent will be summarised. Information regarding the number of patients that are surgically treated versus those who are treated non-operatively will be presented. Data on patient eligibility and reasons for withdrawal from treatment or the trial (when stated) will be summarised. Baseline patient characteristics will be summarized using descriptive statistics; counts for categorical variables and mean/median and inter-quartile range for continuous variables.

General data handling

All analyses will be conducted blinded for the treatment allocation and with pseudonymized data. All statistical tests will be two-sided with an α of 0.05. Primary analyses will be by intention-to-treat. However, per-protocol and as-treated analyses will also be performed. The final pseudonymized dataset will be stored separate from the pseudonymization key on secure hospital and/or university servers, in order to preserve patient confidentiality. forms completed by the patients are delivered at the end of the trial to the central study organisation.

Data analyses

Primary outcome

Equal distribution will be investigated among the two treatment groups for the factors weight-bearing, anticoagulant treatment, time to treatment, surgery vs. no surgery and patient characteristics. The primary outcome will be presented as odds ratios

For peer review only - http://bmjopen.bmj.com/site/about/guidelines.xhtml

and 95% confidence interval. For skewed data, both unadjusted and adjusted results will be presented using logistic regression for the factors weight-bearing, anticoagulant treatment, time to treatment, surgery vs. no surgery and patient characteristics. Absolute reductions in risk of VTE will be calculated from these values, as presented in the reference with similar study design (32).

Secondary outcome

Group differences for ordinal secondary data, such as the patient-reported data, will be analysed by nonparametric tests. Group differences for index scale, ratio scale and continuous secondary outcome will be analysed with appropriate parametric or non-parametric tests. Subgroup analyses will be performed to determine whether certain types of patients might gain greater or less benefit from IPC. The effect of treatment allocation on the primary outcome subdivided by key baseline variables and adjusted for the factors (weight-bearing, anticoagulant treatment, time to treatment, surgery vs. no surgery, compliance and age) will be calculated. Subgroup analyses will be performed by observing the change in log-likelihood when the interaction between the treatment and the subgroup is added into a logistic regression model.

Missing data

Missing data will be investigated and analysed. Reasons for missing data will be stated and summarized where possible. Sensitivity analyses will be performed in order to test the strength and consistency of the final results.

Monitoring/auditing

Research nurses educated in monitoring research studies will perform study monitoring during the trial. Auditing of the trial will be performed by Karolinska Trial Alliance, which is an independent organization from the investigators and sponsor.

For peer review only - http://bmjopen.bmj.com/site/about/guidelines.xhtml

Safety

Risk and side effect

All included patients have an increased risk of developing DVT and PE. There will be no general recommendation for included patients for administrating pharmacoprophylaxis, in line with international guidelines (33, 34). The steering committee advice only to prescribe pharmacoprophylactic drugs to patients with other identifiable risk factors such as previous DVT/PE and usage of birth-control pills. The use of pharmacoprophylaxis will, however, not be an exclusion criterion. Patients with prescription of anticoagulants will be adjusted for in the statistical analysis.

Adverse events and complications

Adverse events in connection with the usage of the intervention (IPC and orthosis) or casting will be noted when reported in the study protocol and referred to the responsible physician if needed. Complications such as infection, fracture dislocation, tendon re-rupture, reoperation or serious life-threatening adverse events will be captured within the clinical injury treatment protocol of the local hospital. Minor or major complications may also be noted through the detailed study protocol and referred to the responsible physician if needed.

Patient and public involvement

A patient representative is on the steering committee of the research project to help in planning and improving the relevance, quality and validity of the research. The patient representative will also receive appropriate training for the job and will actively participate in the development of recommendations and guidelines.

Discussion

Overall aim of the trial

There is an urgent need for more efficient and safer VTE-prophylactics, especially for lower limb immobilized patients, according to major health service authorities (33,

For peer review only - http://bmjopen.bmj.com/site/about/guidelines.xhtml

34). The overall aim of STOP Leg Clots is to investigate whether or not adjuvant intermittent pneumatic compression treatment is reliably effective in outpatient lower leg immobilized patient. We therefore chose two types of injuries, Achilles tendon ruptures and ankle fractures, which constitute a large portion of patients with lower leg immobilization that also have a significantly increased risk of VTE (35). Moreover, patients with ATR and AF have demonstrated low- or non-effectiveness of LMHW prophylaxis also in relation to the risk of bleeding complications (13, 14, 35). In the perspective that different hospitals have different clinical traditions and indications for surgery of ATR and AF we have chosen to include both operated and non-operated ATR/AF, since both groups are equally served by VTE prophylaxis. Earlier studies have shown a just as high incidence of VTE in non-operated as in operated ATR/AF (36), further indicating a benefit of demonstrating a VTE risk reduction in both operated/non-operated lower limb immobilized patients.

Pharmacological prophylaxis

In the initial planning of the trial, the intervention was thought to be compared to patients without any pharmacological prophylaxis in order to study the true VTE preventive effects of the IPC-device. However, there were some sites that did not feel comfortable with this comparator group despite the lack of strong VTE-preventive evidence of pharmacological prophylaxis. In order to ensure a higher inclusion rate into the trial we therefore included the available data for antithrombotic drugs in our power analysis. The primary treating physician will decide if and any prophylactic antithrombotic drugs will be administrated to the patient during the immobilization period, which will be decided prior to randomization. LMWH is the most common drug prescribed for usually 10-14 days, whereas direct oral anticoagulant drugs are seldomly used as pharmacological prophylaxis in previously healthy patients. The trial advices against antithrombotic prophylactic drugs if there are no known risk

For peer review only - http://bmjopen.bmj.com/site/about/guidelines.xhtml

factors for thrombosis and have provided each participating hospital with the international guidelines of whom and when pharmacological treatment should be administered.

Compliance

Compliance to the allocated treatment, specifically number of days and hours of IPC usage, is key in understanding if and how the intervention provide an effect on the primary outcome. This will be monitored by patient self-assessment diaries together with a built-in cumulative timer in the IPC-device. After each week patients will receive an email and be prompted to transfer the data into the REDCap system. American College of Chest Physicians recommend that efforts should be made to achieve 18 h of daily compliance with IPC in hospitalized patients (37). However, there are no recommendations for mobile outpatients. In this study we will recommend at least 10h of bilateral IPC-treatment per 24 hours. However, patients should not be more sedentary than otherwise in order to comply with treatment. Patients will be considered compliant with the intervention if used for at least 30 hours/week throughout the duration of lower limb immobilization.

Endpoints

Compression doppler ultrasonography is the investigation of choice to confirm the diagnosis of DVT since it is non-invasive, acceptable to patients, easily repeated several times and uses equipment widely available (38). Thus consent, accrual and adherence in a trial would more likely be achieved than with alternatives such as phlebography. CDU screening is performed at the end of immobilization, i.e after 6 -8 weeks. CDU screening will detect both symptomatic as well as asymptomatic DVTs. However, it will be difficult to distinguish between the symptomatic and asymptomatic DVTs since calf pain related to DVT can be confounded by the lingering pain from the ankle fracture or Achilles tendon rupture.

For peer review only - http://bmjopen.bmj.com/site/about/guidelines.xhtml

Clinical recommendations

Most DVT's in this trial will be asymptomatic DVT's, only demonstrated by the CDU screening, which will serve as a surrogate outcome for the total VTE burden. The natural history of DVT's seems to be, in the majority of cases, the development of a thrombus in the distal veins of the calf that can extend proximally. However, recent studies demonstrate that even asymptomatic DVT's are associated with reduced patient-reported outcomes one year after initial injury (39) and can extend proximally and cause PE (40). This trial will provide further insight on whether exhibiting a DVT affects patient-reported outcome, as this is one of the secondary outcomes, as well as a cost-benefit analysis of the intervention. The data will also analyse which patients are most likely to exhibit a benefit from the mechanical IPC prophylaxis. If the usage of IPC-devices demonstrates a significant reduction of DVT's we will recommend IPC-devices for prophylactic treatment during lower limb immobilization.

Ethics and dissemination of results

Ethical approval was obtained by the ERB in Stockholm, Sweden, Dnr 2016/1573-31. The results of this study will provide evidence-based treatment protocol for leg immobilized patients. The study results will be submitted for publication to international, peer-reviewed journals, regardless of whether the results are positive, negative or inconclusive in relation to the study hypothesis.

Availability of data and material

The final anonymized data will be available for the PI, the steering-committee and made available upon a reasonable request.

Competing interests

None of the investigators have any financial or other competing interest in relation to the trial. DJO Global is the manufacturer of the IPC-devices used in the study and have made them available for the study sites throughout the study. Disposable

For peer review only - http://bmjopen.bmj.com/site/about/guidelines.xhtml

materials (cuffs and connective tubing) are bought from the DJO Global, Inc. DJO Global has no influence in the conduct of the trial or publication of the results.

Funding

The study is financed by the Swedish research council (Dnr: 2017-00202).
https://www.vr.se/english/about-us/contact.html

Authors' contributions

All authors have contributed to the design of the present trial protocol. PWA is the primary investigator and initiated the study. SS, BA, HB, EDA, KJ and KNH are all helping with study implementation. The protocol was drafted by PWA, SS and refined by BA, HB, EDA, KJ and KNH. SS was responsible for drafting of the manuscript. All authors have read and approved the final manuscript.

Acknowledgements

The STOP Leg Clots trial is currently including patients from the following centers: Akademiska Universitetssjukhuset (Kenneth Jonsson, Catharina Strömstedt), Danderyd Hospital (Erica Domeij-Arverud, Sophia Grindberg, Paula-Therese Kelly, Marie Ax), Gävle Sjukhus (Irina Kolioumpakina Akrioti, Hans Peter Bögl), Helsingborgs lasarett (Anna-Karin Svensson), Höglandssjukhuset Eksjö (Björn Alkner), Karolinska Universitetssjukhuset (Paul Ackermann, Susanna Aufwerber, Luigi Belcastro, Hans Berg, Johanna Flodin, Malin Heijkenskjöld, Simon Svedman, Annukka Saarensilta), Norrtälje Sjukhus (Per-Anton Svensson), Sahlgrenska Universitetssjukhuset (Katarina Nilsson-Helander, Niklas Nilsson), Södersjukhuset (Lasse Lapidus, Elisabeth-Skogman, Annika Tiliander), Uddevalla Sjukhus/Norra Älvsborgs Sjukhus (Anna Ludvigsson, Tina Zorko), Östersunds sjukhus (Harry Mitchell, Alexander De Val Olsson, Jörgen Larsson).

References

1. Cohen AT, Agnelli G, Anderson FA, Arcelus JI, Bergqvist D, Brecht JG, et al. Venous thromboembolism (VTE) in Europe. The number of VTE events and associated morbidity and mortality. Thromb Haemost. 2007;98(4):756-64.

For peer review only - http://bmjopen.bmj.com/site/about/guidelines.xhtml

2. Goldhaber SZ. Venous thromboembolism: epidemiology and magnitude of the problem. Best Pract Res Clin Haematol. 2012;25(3):235-42.
3. Keller K, Tesche C, Gerhold-Ay A, Nickels S, Klok FA, Rappold L, et al. Quality of life and functional limitations after pulmonary embolism and its prognostic relevance. J Thromb Haemost. 2019;17(11):1923-34.
4. Sinha SK, Sachan M, Goel A, Singh K, Mishra V, Jha MJ, et al. Efficacy and Safety of Thrombolytic Therapy in Acute Submassive Pulmonary Embolism: Follow-Up Study. J Clin Med Res. 2017;9(2):163-9.
5. Kahn SR, Shapiro S, Wells PS, Rodger MA, Kovacs MJ, Anderson DR, et al. Compression stockings to prevent post-thrombotic syndrome: a randomised placebo-controlled trial. Lancet. 2014;383(9920):880-8.
6. Makedonov I, Kahn SR, Galanaud JP. Prevention and Management of the Post-Thrombotic Syndrome. J Clin Med. 2020;9(4).
7. Roberts LN, Patel RK, Donaldson N, Bonner L, Arya R. Post-thrombotic syndrome is an independent determinant of health-related quality of life following both first proximal and distal deep vein thrombosis. Haematologica. 2014;99(3):e41-3.
8. Mammen EF. Pathogenesis of venous thrombosis. Chest. 1992;102(6 Suppl):640S-4S.
9. Domeij-Arverud E, Labruto F, Latifi A, Nilsson G, Edman G, Ackermann PW. Intermittent pneumatic compression reduces the risk of deep vein thrombosis during post-operative lower limb immobilisation - a prospective randomised trial of acute ruptures of the Achilles tendon. Bone Joint J 2015;97(B(5)):675-80.
10. Lapidus LJ, Ponzer S, Elvin A, Levander C, Larfars G, Rosfors S, et al. Prolonged thromboprophylaxis with Dalteparin during immobilization after ankle fracture surgery: a randomized placebo-controlled, double-blind study. Acta Orthop. 2007;78(4):528-35.
11. Zee AA, van Lieshout K, van der Heide M, Janssen L, Janzing HM. Low molecular weight heparin for prevention of venous thromboembolism in patients with lower-limb immobilization. Cochrane Database Syst Rev. 2017;8:CD006681.
12. Jorgensen PS, Warming T, Hansen K, Paltved C, Vibeke Berg H, Jensen R, et al. Low molecular weight heparin (Innohep) as thromboprophylaxis in outpatients with a plaster cast: a venografic controlled study. Thrombosis research. 2002;105(6):477-80.
13. Lapidus LJ, Rosfors S, Ponzer S, Levander C, Elvin A, Larfars G, et al. Prolonged thromboprophylaxis with dalteparin after surgical treatment of achilles tendon rupture: a randomized, placebo-controlled study. J Orthop Trauma. 2007;21(1):52-7.
14. Falck-Ytter Y, Francis CW, Johanson NA, Curley C, Dahl OE, Schulman S, et al. Prevention of VTE in orthopedic surgery patients: Antithrombotic Therapy and Prevention of Thrombosis, 9th ed: American College of Chest Physicians Evidence-Based Clinical Practice Guidelines. Chest. 2012;141(2 Suppl):e278S-325S.
15. van Adrichem RA, Nemeth B, Algra A, le Cessie S, Rosendaal FR, Schipper IB, et al. Thromboprophylaxis after Knee Arthroscopy and Lower-Leg Casting. N Engl J Med. 2017;376(6):515-25.
16. Street JT, McGrath M, O'Regan K, Wakai A, McGuinness A, Redmond HP. Thromboprophylaxis using a low molecular weight heparin delays fracture repair. Clin Orthop Relat Res. 2000(381):278-89.
17. Treasure T. Venous thromboembolism: reducing the risk In: Excellence NIfHaC, editor. CG92 ed. London: National Institute for Health and Clinical Excellence; 2010. p. 24-8.
18. utvärdering Sbfmos. Blodpropp - förebyggande, diagnostik och behandling av venös tromboembolism en systematisk kunskapssammanställning [SBU rapport]. Stockholm2002. Available from: https://www.sbu.se/sv/publikationer/SBU-utvarderar/blodpropp---forebyggande-diagnostik-och-behandling-av-venos-tromboembolism/.
19. Gee E. The National VTE Exemplar Centres Network response to implementation of updated NICE guidance: venous thromboembolism in over 16s: reducing the risk of hospital-acquired deep vein thrombosis or pulmonary embolism (NG89). Br J Haematol. 2019;186(5):792-3.

For peer review only - http://bmjopen.bmj.com/site/about/guidelines.xhtml

20. Barrera LM, Perel P, Ker K, Cirocchi R, Farinella E, Morales Uribe CH. Thromboprophylaxis for trauma patients. Cochrane Database Syst Rev. 2013(3):CD008303.
21. Aufwerber S, Edman G, Gravare Silbernagel K, Ackermann PW. Changes in Tendon Elongation and Muscle Atrophy Over Time After Achilles Tendon Rupture Repair: A Prospective Cohort Study on the Effects of Early Functional Mobilization. Am J Sports Med. 2020;48(13):3296-305.
22. Alim MA, Svedman S, Edman G, Ackermann PW. Procollagen markers in microdialysate can predict patient outcome after Achilles tendon rupture. BMJ Open Sport & Exercise Medicine. 2016;2(1).
23. Chan AW, Tetzlaff JM, Altman DG, Laupacis A, Gotzsche PC, Krleza-Jeric K, et al. SPIRIT 2013 statement: defining standard protocol items for clinical trials. Ann Intern Med. 2013;158(3):200-7.
24. Harris PA, Taylor R, Thielke R, Payne J, Gonzalez N, Conde JG. Research electronic data capture (REDCap)--a metadata-driven methodology and workflow process for providing translational research informatics support. J Biomed Inform. 2009;42(2):377-81.
25. Aufwerber S, Heijne A, Edman G, Gravare Silbernagel K, Ackermann PW. Early mobilization does not reduce the risk of deep venous thrombosis after Achilles tendon rupture: a randomized controlled trial. Knee Surg Sports Traumatol Arthrosc. 2020;28(1):312-9.
26. Devlin NJ, Shah KK, Feng Y, Mulhern B, van Hout B. Valuing health-related quality of life: An EQ-5D-5L value set for England. Health Econ. 2018;27(1):7-22.
27. Roos EM, Brandsson S, Karlsson J. Validation of the foot and ankle outcome score for ankle ligament reconstruction. Foot Ankle Int. 2001;22(10):788-94.
28. Nilsson GM, Eneroth M, Ekdahl CS. The Swedish version of OMAS is a reliable and valid outcome measure for patients with ankle fractures. BMC Musculoskelet Disord. 2013;14:109.
29. Nilsson-Helander K, Thomee R, Silbernagel KG, Thomee P, Faxen E, Eriksson BI, et al. The Achilles tendon Total Rupture Score (ATRS): development and validation. Am J Sports Med. 2007;35(3):421-6.
30. Aufwerber S, Heijne A, Gravare Silbernagel K, Ackermann PW. High Plantar Force Loading After Achilles Tendon Rupture Repair With Early Functional Mobilization. Am J Sports Med. 2019;47(4):894-900.
31. Greve K, Domeij-Arverud E, Labruto F, Edman G, Bring D, Nilsson G, et al. Metabolic activity in early tendon repair can be enhanced by intermittent pneumatic compression. Scand J Med Sci Sports. 2012;22(4):e55-63.
32. Collaboration CT, Dennis M, Sandercock P, Reid J, Graham C, Forbes J, et al. Effectiveness of intermittent pneumatic compression in reduction of risk of deep vein thrombosis in patients who have had a stroke (CLOTS 3): a multicentre randomised controlled trial. Lancet. 2013;382(9891):516-24.
33. Falck-Ytter Y, Francis CW, Johanson NA, Curley C, Dahl OE, Schulman S, et al. Prevention of VTE in orthopedic surgery patients: Antithrombotic Therapy and Prevention of Thrombosis, 9th ed: American College of Chest Physicians Evidence-Based Clinical Practice Guidelines. Chest. 2012;141(2 Suppl):e278S-e325S.
34. Treasure T, Hill J. NICE guidance on reducing the risk of venous thromboembolism in patients admitted to hospital. J R Soc Med. 2010;103(6):210-2.
35. Calder JD, Freeman R, Domeij-Arverud E, van Dijk CN, Ackermann PW. Meta-analysis and suggested guidelines for prevention of venous thromboembolism (VTE) in foot and ankle surgery. Knee Surg Sports Traumatol Arthrosc. 2016;24(4):1409-20.
36. Batra S, Kurup H, Gul A, Andrew JG. Thromboprophylaxis following cast immobilisation for lower limb injuries--survey of current practice in United Kingdom. Injury. 2006;37(9):813-7.
37. Fox G, Gabbe BJ, Richardson M, Oppy A, Page R, Edwards ER, et al. Twelve-month outcomes following surgical repair of the Achilles tendon. Injury. 2016;47(10):2370-4.
38. Sampson FC, Goodacre S, Kelly AM, Kerr D. How is deep vein thrombosis diagnosed and managed in UK and Australian emergency departments? Emerg Med J. 2005;22(11):780-2.

For peer review only

39. Svedman S, Westin O, Aufwerber S, Edman G, Nilsson-Helander K, Carmont MR, et al. Longer duration of operative time enhances healing metabolites and improves patient outcome after Achilles tendon rupture surgery. Knee Surg Sports Traumatol Arthrosc. 2018;26(7):2011-20.
40. Krutman M, Kuzniec S, Ramacciotti E, Varella AY, Zlotnik M, Teivelis MP, et al. Rediscussing Anticoagulation in Distal Deep Venous Thrombosis. Clin Appl Thromb Hemost. 2016;22(8):772-8.

For peer review only

Figure. Example template of recommended content for the schedule of enrolment, interventions, and assessments.*

	STUDY PERIOD						
	Enrolment	Allocation	Post-allocation				Close-out
**TIMEPOINT****	*0*		*2w*	*6-8w*	*6m*	*1y*	t_x
**ENROLMENT:**							
**Eligibility screen**	X						
**Informed consent**	X						
**Allocation**		X					
**INTERVENTIONS:**							
Adjuvant IPC treatment	◆———	———	———	———◆			
Standard treatment	◆———	———	———	———◆			
**ASSESSMENTS:**							
Including site	X						
Time of injury	X	X					
Side of injury	X						
Treatment method	X						
Duration of operation	X						
Pharmacoprofylaxis type, dosage, duration	X						
Patient age, length, weight, gender	X						
Patient contact information	X						
Educational level	X						
Current occupation	X						
Yearly salary before taxes	X						

For peer review only

Nicotine usage	X						
Diabetes, hypertension autoimmune disease, inflammatory disease	X						
Post-injury weight-bearing			X	X			
Days of total sickleave				X	X		
Adverse events			X	X		X	
Patient compliance			X	X			
Microdialysis				X			
DVT screening (US)				X			
Lower leg atrophy (CT)				X			
VTE preventive mechanism				X			
Blood-flow quantification				X			
Patient Questionnaire (EQ5D-5L, FAOS, ATRS or OMAS)					X	X	
Heel-rise endurance test (ATR only)						X	
Cost effectiveness analysis						X	

*Recommended content can be displayed using various schematic formats. See SPIRIT 2013 Explanation and Elaboration for examples from protocols.
**List specific timepoints in this row.

Reporting checklist for protocol of a clinical trial.

Based on the SPIRIT guidelines.

Instructions to authors

Complete this checklist by entering the page numbers from your manuscript where readers will find each of the items listed below.

Your article may not currently address all the items on the checklist. Please modify your text to include the missing information. If you are certain that an item does not apply, please write "n/a" and provide a short explanation.

Upload your completed checklist as an extra file when you submit to a journal.

In your methods section, say that you used the SPIRITreporting guidelines, and cite them as:

Chan A-W, Tetzlaff JM, Altman DG, Laupacis A, Gøtzsche PC, Krleža-Jerić K, Hróbjartsson A, Mann H, Dickersin K, Berlin J, Doré C, Parulekar W, Summerskill W, Groves T, Schulz K, Sox H, Rockhold FW, Rennie D, Moher D. SPIRIT 2013 Statement: Defining standard protocol items for clinical trials. Ann Intern Med. 2013;158(3):200-207

		Reporting Item	Page Number
**Administrative information**			
Title	#1	Descriptive title identifying the study design, population, interventions, and, if applicable, trial acronym	1

For peer review only

Trial registration	#2a	Trial identifier and registry name. If not yet registered, name of intended registry	3
Trial registration: data set	#2b	All items from the World Health Organization Trial Registration Data Set	n/a
Protocol version	#3	Date and version identifier	5
Funding	#4	Sources and types of financial, material, and other support	21
Roles and responsibilities: contributorship	#5a	Names, affiliations, and roles of protocol contributors	1, 21
Roles and responsibilities: sponsor contact information	#5b	Name and contact information for the trial sponsor	21
Roles and responsibilities: sponsor and funder	#5c	Role of study sponsor and funders, if any, in study design; collection, management, analysis, and interpretation of data; writing of the report; and the decision to submit the report for publication, including whether they will have ultimate authority over any of these activities	n/a
Roles and responsibilities: committees	#5d	Composition, roles, and responsibilities of the coordinating centre, steering committee, endpoint adjudication committee, data management team, and	21

For peer review only

		other individuals or groups overseeing the trial, if applicable (see Item 21a for data monitoring committee)	
**Introduction**			
Background and rationale	#6a	Description of research question and justification for undertaking the trial, including summary of relevant studies (published and unpublished) examining benefits and harms for each intervention	4
Background and rationale: choice of comparators	#6b	Explanation for choice of comparators	8
Objectives	#7	Specific objectives or hypotheses	7
Trial design	#8	Description of trial design including type of trial (eg, parallel group, crossover, factorial, single group), allocation ratio, and framework (eg, superiority, equivalence, non-inferiority, exploratory)	5
**Methods: Participants, interventions, and outcomes**			
Study setting	#9	Description of study settings (eg, community clinic, academic hospital) and list of countries where data will be collected. Reference to where list of study sites can be obtained	5

For peer review only

Eligibility criteria	#10	Inclusion and exclusion criteria for participants. If applicable, eligibility criteria for study centres and individuals who will perform the interventions (eg, surgeons, psychotherapists)	6, 7
Interventions: description	#11a	Interventions for each group with sufficient detail to allow replication, including how and when they will be administered	7, 8
Interventions: modifications	#11b	Criteria for discontinuing or modifying allocated interventions for a given trial participant (eg, drug dose change in response to harms, participant request, or improving / worsening disease)	n/a
Interventions: adherance	#11c	Strategies to improve adherence to intervention protocols, and any procedures for monitoring adherence (eg, drug tablet return; laboratory tests)	8, 18, 19
Interventions: concomitant care	#11d	Relevant concomitant care and interventions that are permitted or prohibited during the trial	n/a
Outcomes	#12	Primary, secondary, and other outcomes, including the specific measurement variable (eg, systolic blood pressure), analysis metric (eg, change from baseline, final value, time to event), method of aggregation (eg, median, proportion), and time point for each outcome. Explanation of the clinical relevance of chosen efficacy and harm outcomes is strongly recommended	9-12

For peer review only

Participant timeline	#13	Time schedule of enrolment, interventions (including any run-ins and washouts), assessments, and visits for participants. A schematic diagram is highly recommended (see Figure)	12
Sample size	#14	Estimated number of participants needed to achieve study objectives and how it was determined, including clinical and statistical assumptions supporting any sample size calculations	6, 13, 14
Recruitment	#15	Strategies for achieving adequate participant enrolment to reach target sample size	6
**Methods: Assignment of interventions (for controlled trials)**			
Allocation: sequence generation	#16a	Method of generating the allocation sequence (eg, computer-generated random numbers), and list of any factors for stratification. To reduce predictability of a random sequence, details of any planned restriction (eg, blocking) should be provided in a separate document that is unavailable to those who enrol participants or assign interventions	7
Allocation concealment mechanism	#16b	Mechanism of implementing the allocation sequence (eg, central telephone; sequentially numbered, opaque,	7

For peer review only

		sealed envelopes), describing any steps to conceal the sequence until interventions are assigned	
Allocation: implementation	#16c	Who will generate the allocation sequence, who will enrol participants, and who will assign participants to interventions	6-7
Blinding (masking)	#17a	Who will be blinded after assignment to interventions (eg, trial participants, care providers, outcome assessors, data analysts), and how	12
Blinding (masking): emergency unblinding	#17b	If blinded, circumstances under which unblinding is permissible, and procedure for revealing a participant's allocated intervention during the trial	n/a
**Methods: Data collection, management, and analysis**			
Data collection plan	#18a	Plans for assessment and collection of outcome, baseline, and other trial data, including any related processes to promote data quality (eg, duplicate measurements, training of assessors) and a description of study instruments (eg, questionnaires, laboratory tests) along with their reliability and validity, if known. Reference to where data collection forms can be found, if not in the protocol	9-12, 14

For peer review only

Data collection plan: retention	#18b	Plans to promote participant retention and complete follow-up, including list of any outcome data to be collected for participants who discontinue or deviate from intervention protocols	12-13, 18
Data management	#19	Plans for data entry, coding, security, and storage, including any related processes to promote data quality (eg, double data entry; range checks for data values). Reference to where details of data management procedures can be found, if not in the protocol	14-15
Statistics: outcomes	#20a	Statistical methods for analysing primary and secondary outcomes. Reference to where other details of the statistical analysis plan can be found, if not in the protocol	15-16
Statistics: additional analyses	#20b	Methods for any additional analyses (eg, subgroup and adjusted analyses)	16
Statistics: analysis population and missing data	#20c	Definition of analysis population relating to protocol non-adherence (eg, as randomised analysis), and any statistical methods to handle missing data (eg, multiple imputation)	16

Methods: Monitoring

Data monitoring: formal committee	#21a	Composition of data monitoring committee (DMC); summary of its role and reporting structure; statement of whether it is independent from the sponsor and competing interests; and reference to where further	16

For peer review only

		details about its charter can be found, if not in the protocol. Alternatively, an explanation of why a DMC is not needed	
Data monitoring: interim analysis	#21b	Description of any interim analyses and stopping guidelines, including who will have access to these interim results and make the final decision to terminate the trial	N/A
Harms	#22	Plans for collecting, assessing, reporting, and managing solicited and spontaneously reported adverse events and other unintended effects of trial interventions or trial conduct	16-17
Auditing	#23	Frequency and procedures for auditing trial conduct, if any, and whether the process will be independent from investigators and the sponsor	16
**Ethics and dissemination**			
Research ethics approval	#24	Plans for seeking research ethics committee / institutional review board (REC / IRB) approval	3
Protocol amendments	#25	Plans for communicating important protocol modifications (eg, changes to eligibility criteria, outcomes, analyses) to relevant parties (eg, investigators, REC / IRBs, trial participants, trial registries, journals, regulators)	5

For peer review only

Consent or assent	#26a	Who will obtain informed consent or assent from potential trial participants or authorised surrogates, and how (see Item 32)	6
Consent or assent: ancillary studies	#26b	Additional consent provisions for collection and use of participant data and biological specimens in ancillary studies, if applicable	6
Confidentiality	#27	How personal information about potential and enrolled participants will be collected, shared, and maintained in order to protect confidentiality before, during, and after the trial	6
Declaration of interests	#28	Financial and other competing interests for principal investigators for the overall trial and each study site	17-18
Data access	#29	Statement of who will have access to the final trial dataset, and disclosure of contractual agreements that limit such access for investigators	20
Ancillary and post trial care	#30	Provisions, if any, for ancillary and post-trial care, and for compensation to those who suffer harm from trial participation	n/a
Dissemination policy: trial results	#31a	Plans for investigators and sponsor to communicate trial results to participants, healthcare professionals, the public, and other relevant groups (eg, via publication, reporting in results databases, or other data sharing arrangements), including any publication restrictions	20

For peer review only

Dissemination policy: authorship	#31b	Authorship eligibility guidelines and any intended use of professional writers	20
Dissemination policy: reproducible research	#31c	Plans, if any, for granting public access to the full protocol, participant-level dataset, and statistical code	20

Appendices

Informed consent materials	#32	Model consent form and other related documentation given to participants and authorised surrogates	n/a
Biological specimens	#33	Plans for collection, laboratory evaluation, and storage of biological specimens for genetic or molecular analysis in the current trial and for future use in ancillary studies, if applicable	10-11

None The SPIRIT checklist is distributed under the terms of the Creative Commons Attribution License CC-BY-ND 3.0. This checklist can be completed online using https://www.goodreports.org/, a tool made by the EQUATOR Network in collaboration with Penelope.ai

For peer review only

BMJ Open

STOP Leg Clots - Swedish multicenter Trial of Outpatient Prevention of Leg Clots: study protocol for a randomized controlled trial on the efficacy of intermittent pneumatic compression on venous thromboembolism in lower leg immobilized patients

Journal:	*BMJ Open*
Manuscript ID	bmjopen-2020-044103.R2
Article Type:	Protocol
Date Submitted by the Author:	13-Apr-2021

Complete List of Authors:	Svedman, Simon; Karolinska Institutet, Molecular Medicine and Surgery; Karolinska University Hospital, Department of Orthopedic Surgery<br>Alkner, Björn; Linköping University, Department of Orthopaedics, Eksjö, Region Jönköping County and Department of Biomedical and Clinical Sciences<br>Berg, Hans; Karolinska University Hospital, Department of Orthopedic Surgery; Karolinska institutet Department of Clinical Sciences Intervention and Technology, Division of Orthopaedics and Biotechnology<br>Domeij-Arverud, Erica; Karolinska Institutet, Department och clinical sciences, Danderyd Hospital<br>Jonsson, Kenneth; Uppsala University Hospital, Department of Surgical Sciences<br>Nilsson Helander, Katarina; Sahlgrenska University Hospital, Department of Orhopaedics<br>Ackermann, Paul; Karolinska Institutet, Department of Molecular Medicine and Surgery; Karolinska University Hospital, Department of Orthopedic Surgery

**Primary Subject Heading**:	Sports and exercise medicine
Secondary Subject Heading:	Haematology (incl blood transfusion)
Keywords:	Foot & ankle < ORTHOPAEDIC & TRAUMA SURGERY, Thromboembolism < CARDIOLOGY, Anticoagulation < HAEMATOLOGY, Orthopaedic sports trauma < ORTHOPAEDIC & TRAUMA SURGERY, Ultrasound < RADIOLOGY & IMAGING, Orthopaedic & trauma surgery < SURGERY

SCHOLARONE™
Manuscripts

I, the Submitting Author has the right to grant and does grant on behalf of all authors of the Work (as defined in the below author licence), an exclusive licence and/or a non-exclusive licence for contributions from authors who are: i) UK Crown employees; ii) where BMJ has agreed a CC-BY licence shall apply, and/or iii) in accordance with the terms applicable for US Federal Government officers or employees acting as part of their official duties; on a worldwide, perpetual, irrevocable, royalty-free basis to BMJ Publishing Group Ltd ("BMJ") its licensees and where the relevant Journal is co-owned by BMJ to the co-owners of the Journal, to publish the Work in this journal and any other BMJ products and to exploit all rights, as set out in our licence.

The Submitting Author accepts and understands that any supply made under these terms is made by BMJ to the Submitting Author unless you are acting as an employee on behalf of your employer or a postgraduate student of an affiliated institution which is paying any applicable article publishing charge ("APC") for Open Access articles. Where the Submitting Author wishes to make the Work available on an Open Access basis (and intends to pay the relevant APC), the terms of reuse of such Open Access shall be governed by a Creative Commons licence – details of these licences and which Creative Commons licence will apply to this Work are set out in our licence referred to above.

Other than as permitted in any relevant BMJ Author's Self Archiving Policies, I confirm this Work has not been accepted for publication elsewhere, is not being considered for publication elsewhere and does not duplicate material already published. I confirm all authors consent to publication of this Work and authorise the granting of this licence.

For peer review only

STOP Leg Clots - Swedish multicenter Trial of Outpatient Prevention of Leg Clots: study protocol for a randomized controlled trial on the efficacy of intermittent pneumatic compression on venous thromboembolism in lower leg immobilized patients

Simon Svedman[1,2], Björn A. Alkner[3], Hans E Berg[2,4], Erica Domeij-Arverud[5], Kenneth B. Jonsson[6], Katarina Nilsson Helander[7], Paul W. Ackermann[1,2]

1 - Department of Molecular Medicine and Surgery, Karolinska Institutet, Stockholm, Sweden.

2 - Department of Orthopaedic Surgery, Karolinska University Hospital, Stockholm, Sweden

3 - Department of Orthopaedics, Eksjö, Region Jönköping County and Department of Biomedical and Clinical Sciences, Linköping University, Linköping, Sweden

4 - Division of Orthopaedics and Biotechnology, Department of Clinical Science, Intervention and Technology, Karolinska Institutet, Stockholm, Sweden

5 - Karolinska Institutet, Department of Clinical Sciences, Danderyd Hospital, Stockholm, Sweden.

6 - Department of Surgical Sciences, Uppsala University Hospital, Uppsala, Sweden

7 - Department of Orthopaedics, Sahlgrenska University Hospital, Gothenburg, Sweden

Email

Simon.svedman@ki.se, bjorn.alkner@liu.se, hans.er.berg@sll.se, Erica.domeij-arverud@sll.se, kenneth.jonsson@surgsci.uu.se, katarina.nilsson.helander@vgregion.se, paul.ackermann@ki.se

Corresponding author:

Paul W. Ackermann;
Karolinska Universitetssjukhuset, Ortopedmottagningen A6:01, 171 76 Stockholm
Fax: +46851772695, Phone: +46851771734, Email: Paul.ackermann@ki.se

For peer review only - http://bmjopen.bmj.com/site/about/guidelines.xhtml

Abstract:

Introduction:

Leg immobilization in a cast or an orthosis after lower limb injuries is associated with a high risk of complications of venous thromboembolism (VTE) and hampered healing. Current pharmacoprophylaxes of VTE are inefficient and associated with adverse events. Intermittent pneumatic compression (IPC) could represent a novel, efficient and safe VTE-prophylactic alternative that may enhance injury healing. The aim of STOP leg clots is to assess the efficacy of adjuvant IPC-therapy on reduction of VTE-incidence and improvement of healing in lower leg immobilized outpatients.

Methods and analysis:

STOP leg clots is a multicentre randomised controlled superiority trial. Eligible patients (700 patients/arm) with either an acute ankle fracture or Achilles tendon rupture will be randomized to either addition of IPC during lower-leg immobilization or to treatment-as-usual. The primary outcome will be the total VTE-incidence, i.e. symptomatic and asymptomatic deep venous thrombosis (DVT) or symptomatic pulmonary embolism (PE), during the leg immobilization period, approximately 6-8 weeks. DVT-incidence will be assessed by screening whole leg compression duplex ultrasound at removal of leg immobilization and/or clinically diagnosed within the time of immobilization. Symptomatic PE will be verified by computer tomography.

Secondary outcomes will include patient-reported outcome using validated questionnaires, healing evaluated by measurements of tendon callus production and changes in VTE-prophylactic mechanisms assessed by blood flow and fibrinolysis. Data analyses will be blinded and based on the intention-to-treat.

For peer review only

Ethics and dissemination:

Ethical approval was obtained by the ethical review board in Stockholm, Sweden, Dnr 2016/1573-31. The study will be conducted in accordance with the Helsinki declaration. The results of the study will be disseminated in peer-reviewed international journals.

Trial Registration:

ClinicalTrials.gov Identifier: NCT03259204, registered August 23, 2017.

Time Schedule

01-09-2018 – 31-12-2022

Strengths and limitations of this study

- This is the first multicentre randomized controlled trial to investigate the efficiency of adjuvant IPC on VTE for the duration of ankle joint immobilization in an outpatient setting.
- The multicenter design improves the external validity of the study and will allow rapid dissemination and potential implementation of study results.
- Patients cannot be blinded to allocation.
- Clinical sonographers are responsible to assess the primary outcome and will be blinded to patient allocation.
- The duplex ultrasound screening will detect subclinical VTE which will allow high study power. However, the clinical relevance of non-symptomatic events is still not fully clear.

For peer review only

Introduction

Venous thromboembolism (VTE), comprising deep venous thrombosis (DVT) and pulmonary embolism (PE), is a critical medical condition and potentially deadly. An estimated 370.000 people die of VTE in Europe each year, and more than 700.000 Europeans are afflicted(1). Patients who survive often suffer chronic ailments with significantly lower quality of life after an event (2-7). Leg immobilization in a cast or orthosis is a routine stage in the treatment of patients with lower limb injury, which however causes high rates of VTE due to venous stasis (8). Estimated DVT incidence may range from 21% up to 50% after ankle fractures (AF) and Achilles tendon ruptures (ATR), respectively (9, 10).

The most widely used method to prevent VTE during lower leg immobilization is by use of pharmacological prophylaxis, such as low molecular weight heparin (LMWH). However, several studies have demonstrated only a marginal or no effect of LMWH in lower leg immobilized patients and LMWH is therefore not cost efficient for these patients (7, 11-15). In addition to their low efficacy, LMWH increases the risk of bleeding complications (13, 14) and may impair tissue healing (16). For these reasons, the National Health Service in UK, and the corresponding authority in Sweden, have stressed the need for safer and more efficient VTE-prophylaxis (17-19).

Mechanical thromboprophylaxis with intermittent pneumatic compression (IPC) reduces VTE, is a cost-effective intervention in hospitalized patients, and is recommended by guidelines for surgical in-hospital patients (17, 20). Whether long-term IPC treatment applied during leg immobilization in an outpatient setting can reduce the incidence of DVT is unknown. We recently published a study presenting

For peer review only - http://bmjopen.bmj.com/site/about/guidelines.xhtml

preliminary evidence that leg immobilized patients with ATR treated with adjuvant IPC at 2 weeks had a reduced DVT rate (21% vs. 37%) compared to controls using Doppler ultrasound screening. However, in the previous study the IPC intervention ended at two weeks and at 6 weeks the DVT-rate was 50% in both groups (9).

Thus, the primary aim of this prospective, multicentre, randomized controlled trial is to evaluate the efficacy of prolonged adjuvant IPC therapy in leg-immobilized patients on the total incidence of VTE up to removal of leg immobilization.

Lower leg immobilization, in addition to increased risk of VTE, may also lead to impaired healing after initial injury as well as long lasting muscle atrophy (21). As recent studies suggest that the increased neuro-vascular flow induced by IPC could trigger the healing process (22), we also aim to assess whether adjuvant IPC enhances healing, reduces muscle atrophy and improves the long-term patient-reported outcomes in lower limb immobilized patients.

Methods and analysis

This is a report of the trial protocol dated 4th September 2017, supported by the Swedish research council. Any important protocol modifications will be communicated through ClinicalTrials.gov trial number NCT03259204, the Ethical Review Board and/or the BMJ Open journal. The protocol was developed in accordance with Standard Protocol Items: Recommendations for Interventional Trials and Template for Intervention Description and Replication statements (23).

Study design and setting

“STOP leg clots” is a multicentre, prospective, superiority, randomized, controlled, parallel group trial with blinded assessment of the outcome.

For peer review only - http://bmjopen.bmj.com/site/about/guidelines.xhtml

For peer review only

Recruitment organization

In general, patients will be informed about the study at their visit to the emergency department. As this is a multicentre trial, different methods will be suitable and used by different participating study-sites in order to include patients. Patients who are not subjected to further treatment in the hospital will either be included and randomized immediately after given both written and oral information about the study, or if this is not possible, receive information about the study and scheduled for an outpatient study appointment within the following days. Patients who are in need of surgery and operated on within 10 days can be included and randomized, after receiving written and oral information. All participating patients will sign an informed consent form, which will be collected by the local study personnel and delivered to the central study organization for archiving and the end of the trial. The steering committee will aid recruiting centres in setting up the local infra-structure needed for patient recruitment.

Study Organization

11 hospitals from 7 different Swedish regions and 2 hospitals from 2 other European countries have so far accepted to participate in the study.

Study participants

1400 patients will be included; whereof approximately 1000 ankle fractures and 400 Achilles tendon ruptures.

- Inclusion criteria: age of 18-75 years, unilateral isolated ankle fracture or Achilles tendon rupture and able to be included in the study within 10 days of injury.

- Exclusion criteria: unable to give informed consent, unable to follow instructions and unable to receive orthosis treatment, known kidney failure, heart failure with pitting oedema, malignancy, haemophilia, thrombophilia,

For peer review only

current DVT or PE, pregnancy, or has follow-up planned at a hospital outside of the study organization.

Objective

The primary objective is to investigate if adjuvant IPC treatment during the duration of lower leg immobilization treatment will reduce the incidence of VTE in an outpatient setting.

Randomization

Patients will prior to randomization receive both written and oral information and sign an informed consent form. Patients will be randomly assigned to either standard treatment or standard treatment with adjuvant IPC in an orthosis with a 1:1 allocation. Patient randomization may occur up to 10 days after the injury. The number of days from injury to start of randomized treatment as well as potential immobilization will be noted. The randomization is stratified by study site, patient age over/under 40 years and by type of injury (AF or ATR). Randomization occurs in blocks of 4. Randomization lists are uploaded to the REDCap online eCRF system (24) hosted at Karolinska Institutet, only accessible to the REDCap administrator of the trial. Randomization is only performed by instructed study personnel. All activities within REDCap are tracked, and the REDCap log is non-modifiable.

Intervention

The intervention, *addition of IPC to routine care*, will be administered in the emergency department, the outpatient clinic or the hospital ward. Patients randomized to the intervention will during the whole duration of lower limb immobilization receive bilateral calf-IPC (Venaflow® Elite, DJO LLC, Vista, CA, USA), on the injured side applied under an orthosis (Aircast Airselect™ Elite Walker, DJO LLC, Vista, CA, USA). Calf-IPC has previously been successfully used under an orthosis (9). Patients wearing an orthosis are instructed to open the orthosis 2-3

For peer review only - http://bmjopen.bmj.com/site/about/guidelines.xhtml

times per day while sedentary to reduce the humidity in the orthosis and prevent blistering. When IPC is used, the calf-cuffs will alternately inflate every 30 seconds. The patients will be instructed to apply the IPC-therapy as much as possible during the time they are sedentary, both day and night, with a goal of 10 h/24 h. Patients should, however, not keep themselves from being active in order to comply with IPC-treatment. Compliance with the treatment will be registered by both the patient and by the device (9). IPC will be discontinued if the patient declines to continue IPC or had adverse effects of the IPC that warrants removal. Patients that use the IPC for 30 hours/week or more will be deemed compliant to the treatment. IPC-treatment will be ended when the leg immobilization is removed.

Comparator

Patients randomized to standard care will receive lower limb immobilization in an orthosis or a below-knee plaster cast according to local routines. A previous study did not show any difference in the incidence of VTE between plaster cast immobilization and orthosis-treatment (25). As standard care varies according to local routines and type of injury regarding duration of immobilization as well as weight-bearing these factors will be recorded and analysed for.

Intake of pharmacological antithrombotic drugs before the injury or administered during the immobilization period will be registered for category, dosage and duration. There is no general recommendation for prescribing VTE-prophylactic drugs in the trial for patients without any known risk factors for thrombosis.

Study outcomes

Primary Outcome measure

The primary outcome is the incidence of VTE during the lower leg immobilized period. VTE will be assessed clinically if a patient develops symptoms of DVT or PE,

For peer review only - http://bmjopen.bmj.com/site/about/guidelines.xhtml

which is verified by compression Duplex ultrasound or computer tomography, respectively. Moreover, DVT will be screened for by whole leg CDU of the injured leg at termination of leg immobilization by an experienced sonographer, which is 6-8 weeks after injury, depending on the type of injury and treatment used. The patient will furthermore be asked to answer if a symptomatic VTE has occurred after the initial 6-8 week follow-up in the survey at 12 months.

Secondary outcome measure

Secondary outcome planned for the study are listed below. Many of the secondary outcome measures need a much smaller number of patients included and will therefore only be performed on a limited number of sites to decrease the risk of methodological errors.

Secondary outcome measures include:

- Patient Reported Outcome Measures (PROM) will be reported by all participating patients at 6- and 12 months after injury. The questionnaires EQ-5D-5L (26) and the Foot- and Ankle Outcome Score (FAOS) (27) will be answered by all patients. In addition, the Olerud-Molander Outcome Score (OMAS) (28) will be answered by the ankle fracture patients and the Achilles tendon Total Rupture Score (ATRS) (29) by the Achilles tendon rupture patients. The data will be collected and managed using REDCap electronic data capture tool (24).
- Compliance to IPC-treatment. Patients randomized to IPC-treatment will give weekly reports regarding IPC-device usage by daily journals. The IPC-device will also record the cumulative time used.
- Post-injury weight-bearing. Weight-bearing after injury have been suggested to improve lower limb-blood flow and decrease risk of DVT (25). Weight-bearing

For peer review only - http://bmjopen.bmj.com/site/about/guidelines.xhtml

will be recorded via patient recorded daily journals. The patient is asked to note how much weight they load on their injured foot while walking, on a scale from 0-100. 0 is equal to non-weight-bearing walking with the aid of crutches and 100 is fully weight-bearing in the orthosis/plaster cast on the injured foot without crutches. A recent study has shown that this scale analysis is a reliable method of assessing weight-bearing of the patient (30).

- Adverse events. Events such as post-operative infection, fracture dislocation, re-rupture or re-operation will be recorded. Furthermore, patient mortality will be registered at one year after injury.
- IPC-related adverse events. Events caused by IPC-treatment such as skin damage or infection, prematurely discontinuing IPC-treatment or fall injuries will be recorded.
- Microdialysis of Achilles tendon healing. Healing metabolites as well as callus production will be assessed at termination of leg immobilization, 6-8 weeks, using microdialysis in a subgroup of 50 ATR patients, followed by quantification of procollagens, to assess tendon healing. Microdialysis is a well-known process for this aim, where a small catheter is inserted in the paratenon space (31). Samples will be collected and stored at Stockholm medical biobank.
- Heel-rise endurance test. The heel-rise endurance test is an established functional test for patients with Achilles tendon ruptures and will be performed by a subgroup of patients at 1 year after injury, to measure muscle-tendon strength in the injured, compared to the uninjured leg. The MuscleLab (Ergo Test Technology, Stathelle, Norway) measurement system is used.

For peer review only - http://bmjopen.bmj.com/site/about/guidelines.xhtml

- VTE-preventive mechanisms. Assessment of fibrinolysis and coagulation factors as well as blood-flow quantification using ultrasound will be assessed in a subgroup of 50 patients. Overall hemostatic potential, D-dimer, Plasminogen activator inhibitor-1, endogen thrombin potential, trombin-antitrombin-complex, plasmin antiplasmin complex and tissue plasminogen activator/PAI complex will be assessed at Karolinska Institutet, Coagulation Unit. Samples will be collected from patients willing to participate at discontinuation of lower limb immobilization, prepared and stored at Stockholm medical biobank until analysis.
- The incidence of VTE in patients using anticoagulants such as low molecular weight heparin (LMWH) with or without adjuvant IPC will be analysed.
- Blood-flow quantification. Peak popliteal and femoral blood velocity will be calculated using custom ultrasound software and compared with baseline values, while microcirculation and tissue oxygenation will be assessed by near-infrared spectroscopy (INVOS®). These explanatory continuous data will be compared between the groups.
- Cost-benefit analysis. Patient annual salary, type of occupation and the numbers of days on sick-leave will be recorded. These data will be used in calculating cost-effectiveness for the prevention of VTE.

Blinding

Blinding will not be possible for the patient or for the study personnel responsible for patient inclusion. Sonographers will be blinded to patient allocation since the immobilization will be removed prior to ultrasound examination. All patients are instructed not to disclose randomization allocation to the sonographers, and the sonographers are informed not to ask about this information.

For peer review only

Time Schedule

Recruitment of patients began September 1, 2018. Estimated primary completion date is December 31 2022.

Setup

Follow-up investigations

All patients will be followed as usual with an outpatient visit approximately at 2 weeks after injury/surgery where compliance and adverse events will be recorded. At the time of termination of treatment (usually 6-8 weeks after injury/surgery) the orthosis will be removed and the patient will be examined by an orthopaedic surgeon or a physiotherapist, who will document compliance and adverse events. At this time the ultrasound investigation of the lower limbs will occur, following a standardized protocol. The patient will be treated according to local routines in the event of a venous thrombosis.

Patients will receive a journal where they will note the estimated amount of loading on their injured leg (0-100%) and, if randomized to intervention, how many hours they used IPC-treatment each day. This will be self-reported via the REDCap digital clinical report forms once a week and re-checked at the last out-patient when discontinuing the lower leg immobilization. Patients who do not have access to an e-mail address or do not want to report their data digitally, will hand in their journal at the discontinuation of immobilization, and the data will be entered manually by the research personnel.

Protocol for duplex ultrasound

Ultrasound will be performed throughout by trained sonographers on the injured leg only. Locations investigated include the deep veins (femoralis communis, femoralis superficialis, femoralis profunda, poplitea, tibialis posterior and fibularis) and muscular veins. A positive investigation is reported when any of the following is

For peer review only

observed: visible thrombi in the 2D projection, a non-compressible vein, an expanded vein and lack of doppler signal or stripe flow. All positive findings will be validated by a sonographer or radiologist in the steering-committee.

Statistical analysis

Sample size

The STOP Leg Clots is designed on a hypothesis of superiority of adjuvant intermittent pneumatic compression therapy compared to standard treatment during lower leg immobilization in prevention of venous thromboembolism. Based on the existing literature the expected occurrence of DVT in the control group as detected by screening ultrasound is up to 25% for AF and 50% in ATR (9, 10). The estimates are dependent on the case-mix ratio as the incidence of DVT is higher in ATR than in AF. We expect the case-mix to be 30% ATR and 70% AF, resulting in an expected DVT-rate of 33%. As extra precautions we reduced the expected total DVT-rate from 33% to 28%. The expected relative risk reduction of DVT is 40% based on earlier studies (32), and we chose to calculate with a lower risk reduction value of 29%. This leads to an absolute risk reduction of DVT of 8%, which is higher than the suggested minimum clinically relevant absolute risk reduction of 4% (9, 32). The sample size calculations for the study are based on the following estimates and parameters: alpha = 0.05, 1-beta = 90%, estimated incidence of DVT = 28%, the smallest relative risk reduction to detect= 29%. The sample size needed using these study parameters is 597 in each group. Expecting a drop-out rate of 5-10% and to compensate for the uncertainty of the final case-mix distribution we aim to include 1400 patients in our study, around 1000 AF patients and 400 ATR patients.

For peer review only - http://bmjopen.bmj.com/site/about/guidelines.xhtml

Statistical analysis plan

Descriptive statistics

The flow of patients through the trial will be displayed according to the CONSORT statement. The number of patients screened; number of ineligible patients and the reasons why; number of eligible patients not providing consent; and the number of eligible patients subsequently randomised will be presented. The characteristics of the screened population, the ineligible participants and eligible participants who consent and do not consent will be summarised. Information regarding the number of patients that are surgically treated versus those who are treated non-operatively will be presented. Data on patient eligibility and reasons for withdrawal from treatment or the trial (when stated) will be summarised. Baseline patient characteristics will be summarized using descriptive statistics; counts for categorical variables and mean/median and inter-quartile range for continuous variables.

General data handling

All analyses will be conducted blinded for the treatment allocation and with pseudonymized data. All statistical tests will be two-sided with an α of 0.05. Primary analyses will be by intention-to-treat. However, per-protocol and as-treated analyses will also be performed. The final pseudonymized dataset will be stored separate from the pseudonymization key on secure hospital and/or university servers, in order to preserve patient confidentiality. Forms completed by the patients are delivered at the end of the trial to the central study organisation.

Data analyses

Primary outcome

Equal distribution will be investigated among the two treatment groups for the factors weight-bearing, anticoagulant treatment, time to treatment, surgery vs. no surgery and patient characteristics. The primary outcome will be presented as odds ratios

For peer review only - http://bmjopen.bmj.com/site/about/guidelines.xhtml

and 95% confidence interval. For skewed data, both unadjusted and adjusted results will be presented using logistic regression for the factors weight-bearing, anticoagulant treatment, time to treatment, surgery vs. no surgery and patient characteristics. Absolute reductions in risk of VTE will be calculated from these values, as presented in the reference with similar study design (32).

Secondary outcome

Group differences for ordinal secondary data, such as the patient-reported data, will be analysed by nonparametric tests. Group differences for index scale, ratio scale and continuous secondary outcome will be analysed with appropriate parametric or non-parametric tests. Subgroup analyses will be performed to determine whether certain types of patients might gain greater or less benefit from IPC. The effect of treatment allocation on the primary outcome subdivided by key baseline variables and adjusted for the factors (weight-bearing, anticoagulant treatment, time to treatment, surgery vs. no surgery, compliance and age) will be calculated. Subgroup analyses will be performed by observing the change in log-likelihood when the interaction between the treatment and the subgroup is added into a logistic regression model.

Missing data

Missing data will be investigated and analysed. Reasons for missing data will be stated and summarized where possible. Sensitivity analyses will be performed in order to test the strength and consistency of the final results.

Monitoring/auditing

Research nurses educated in monitoring research studies will perform study monitoring during the trial. Auditing of the trial will be performed by Karolinska Trial Alliance, which is an independent organization from the investigators and sponsor.

For peer review only - http://bmjopen.bmj.com/site/about/guidelines.xhtml

Safety

Risk and side effect

All included patients have an increased risk of developing DVT and PE. There will be no general recommendation for included patients for administrating pharmacoprophylaxis, in line with international guidelines (33, 34). The steering committee advice only to prescribe VTE-prophylactic drugs to patients with other identifiable risk factors such as previous DVT/PE and usage of birth-control pills. The use of pharmacoprophylaxis will, however, not be an exclusion criterion. Patients with prescription of anticoagulants will be adjusted for in the statistical analysis.

Adverse events and complications

Adverse events in connection with the usage of the intervention (IPC and orthosis) or casting will be noted when reported in the study protocol and referred to the responsible physician if needed. Complications such as infection, fracture dislocation, tendon re-rupture, reoperation or serious life-threatening adverse events will be captured within the clinical injury treatment protocol of the local hospital. Minor or major complications may also be noted through the detailed study protocol and referred to the responsible physician if needed.

Patient and public involvement

A patient representative is on the steering committee of the research project to help in planning and improving the relevance, quality and validity of the research. The patient representative will also receive appropriate training for the job and will actively participate in the development of recommendations and guidelines.

Discussion

Overall aim of the trial

There is an urgent need for more efficient and safer VTE-prophylactics, especially for lower limb immobilized patients, according to major health service authorities (33,

For peer review only - http://bmjopen.bmj.com/site/about/guidelines.xhtml

34). The overall aim of STOP Leg Clots is to investigate whether or not adjuvant intermittent pneumatic compression treatment is reliably effective in outpatient lower leg immobilized patient. We therefore chose two types of injuries, Achilles tendon ruptures and ankle fractures, which constitute a large portion of patients with lower leg immobilization that also have a significantly increased risk of VTE (35). Moreover, patients with ATR and AF have demonstrated low- or non-effectiveness of LMHW prophylaxis also in relation to the risk of bleeding complications (13, 14, 35). In the perspective that different hospitals have different clinical traditions and indications for surgery of ATR and AF we have chosen to include both operated and non-operated ATR/AF, since both groups are equally served by VTE prophylaxis. Earlier studies have shown a just as high incidence of VTE in non-operated as in operated ATR/AF (36), further indicating a benefit of demonstrating a VTE risk reduction in both operated/non-operated lower limb immobilized patients.

Pharmacological prophylaxis

In the initial planning of the trial, the intervention was thought to be compared to patients without any pharmacological prophylaxis in order to study the true VTE preventive effects of the IPC-device. However, there were some sites that did not feel comfortable with this comparator group despite the lack of strong VTE-preventive evidence of pharmacological prophylaxis. In order to ensure a higher inclusion rate into the trial we therefore included the available data for antithrombotic drugs in our power analysis. The primary treating physician will decide if and any prophylactic antithrombotic drugs will be administrated to the patient during the immobilization period, which will be decided prior to randomization. LMWH is the most common drug prescribed for usually 10-14 days, whereas direct oral anticoagulant drugs are seldomly used as pharmacological prophylaxis in previously healthy patients. The trial advices against antithrombotic prophylactic drugs if there are no known risk

For peer review only - http://bmjopen.bmj.com/site/about/guidelines.xhtml

factors for thrombosis and have provided each participating hospital with the international guidelines of whom and when pharmacological treatment should be administered.

Compliance

Compliance to the allocated treatment, specifically number of days and hours of IPC usage, is key in understanding if and how the intervention provide an effect on the primary outcome. This will be monitored by patient self-assessment diaries together with a built-in cumulative timer in the IPC-device. After each week patients will receive an email and be prompted to transfer the data into the REDCap system. American College of Chest Physicians recommend that efforts should be made to achieve 18 h of daily compliance with IPC in hospitalized patients (37). However, there are no recommendations for mobile outpatients. In this study we will recommend at least 10h of bilateral IPC-treatment per 24 hours. However, patients should not be more sedentary than otherwise in order to comply with treatment. Patients will be considered compliant with the intervention if used for at least 30 hours/week throughout the duration of lower limb immobilization.

Endpoints

Compression doppler ultrasonography is the investigation of choice to confirm the diagnosis of DVT since it is non-invasive, acceptable to patients, easily repeated several times and uses equipment widely available (38). Thus consent, accrual and adherence in a trial would more likely be achieved than with alternatives such as phlebography. CDU screening is performed at the end of immobilization, i.e after 6 -8 weeks. CDU screening will detect both symptomatic as well as asymptomatic DVTs. However, it will be difficult to distinguish between the symptomatic and asymptomatic DVTs since calf pain related to DVT can be confounded by the lingering pain from the ankle fracture or Achilles tendon rupture.

For peer review only - http://bmjopen.bmj.com/site/about/guidelines.xhtml

Clinical recommendations

Most DVT's in this trial will be asymptomatic DVT's, only demonstrated by the CDU screening, which will serve as a surrogate outcome for the total VTE burden. The natural history of DVT's seems to be, in the majority of cases, the development of a thrombus in the distal veins of the calf that can extend proximally. However, recent studies demonstrate that even asymptomatic DVT's are associated with reduced patient-reported outcomes one year after initial injury (39) and can extend proximally and cause PE (40). This trial will provide further insight on whether exhibiting a DVT affects patient-reported outcome, as this is one of the secondary outcomes, as well as a cost-benefit analysis of the intervention. The data will also be analysed to give results on which patients are most likely to exhibit a benefit from the mechanical IPC prophylaxis. If the usage of IPC-devices demonstrates a significant reduction of DVT's we will recommend IPC-devices for prophylactic treatment during lower limb immobilization.

Ethics and dissemination of results

Ethical approval was obtained by the ERB in Stockholm, Sweden, Dnr 2016/1573-31. The results of this study will provide evidence-based treatment protocol for leg immobilized patients. The study results will be submitted for publication to international, peer-reviewed journals, regardless of whether the results are positive, negative or inconclusive in relation to the study hypothesis.

Availability of data and material

The final anonymized data will be available for the PI, the steering-committee and made available upon a reasonable request.

Competing interests

None of the investigators have any financial or other competing interest in relation to the trial. DJO Global is the manufacturer of the IPC-devices used in the study and

For peer review only

have made them available for the study sites throughout the study. Disposable materials (cuffs and connective tubing) are bought from the DJO Global, Inc. DJO Global has no influence in the conduct of the trial or publication of the results.

Funding

The study is financed by the Swedish research council (Dnr: 2017-00202). https://www.vr.se/english/about-us/contact.html

Authors' contributions

All authors have contributed to the design of the present trial protocol. PWA is the primary investigator and initiated the study. SS, BA, HB, EDA, KJ and KNH are all helping with study implementation. The protocol was drafted by PWA, SS and refined by BA, HB, EDA, KJ and KNH. SS was responsible for drafting of the manuscript. All authors have read and approved the final manuscript.

Acknowledgements

The STOP Leg Clots trial is currently including patients from the following centers: Akademiska Universitetssjukhuset (Kenneth Jonsson, Catharina Strömstedt), Danderyd Hospital (Erica Domeij-Arverud, Sophia Grindberg, Paula-Therese Kelly, Marie Ax), Gävle Sjukhus (Irina Kolioumpakina Akrioti, Hans Peter Bögl), Helsingborgs lasarett (Anna-Karin Svensson), Höglandssjukhuset Eksjö (Björn Alkner), Karolinska Universitetssjukhuset (Paul Ackermann, Susanna Aufwerber, Luigi Belcastro, Hans Berg, Johanna Flodin, Malin Heijkenskjöld, Simon Svedman, Annukka Saarensilta), Norrtälje Sjukhus (Per-Anton Svensson), Sahlgrenska Universitetssjukhuset (Katarina Nilsson-Helander, Niklas Nilsson), Södersjukhuset (Lasse Lapidus, Elisabeth-Skogman, Annika Tiliander), Uddevalla Sjukhus/Norra Älvsborgs Sjukhus (Anna Ludvigsson, Tina Zorko), Östersunds sjukhus (Harry Mitchell, Alexander De Val Olsson, Jörgen Larsson).

For peer review only

References

1. Cohen AT, Agnelli G, Anderson FA, Arcelus JI, Bergqvist D, Brecht JG, et al. Venous thromboembolism (VTE) in Europe. The number of VTE events and associated morbidity and mortality. Thromb Haemost. 2007;98(4):756-64.
2. Goldhaber SZ. Venous thromboembolism: epidemiology and magnitude of the problem. Best Pract Res Clin Haematol. 2012;25(3):235-42.
3. Keller K, Tesche C, Gerhold-Ay A, Nickels S, Klok FA, Rappold L, et al. Quality of life and functional limitations after pulmonary embolism and its prognostic relevance. J Thromb Haemost. 2019;17(11):1923-34.
4. Sinha SK, Sachan M, Goel A, Singh K, Mishra V, Jha MJ, et al. Efficacy and Safety of Thrombolytic Therapy in Acute Submassive Pulmonary Embolism: Follow-Up Study. J Clin Med Res. 2017;9(2):163-9.
5. Kahn SR, Shapiro S, Wells PS, Rodger MA, Kovacs MJ, Anderson DR, et al. Compression stockings to prevent post-thrombotic syndrome: a randomised placebo-controlled trial. Lancet. 2014;383(9920):880-8.
6. Makedonov I, Kahn SR, Galanaud JP. Prevention and Management of the Post-Thrombotic Syndrome. J Clin Med. 2020;9(4).
7. Roberts LN, Patel RK, Donaldson N, Bonner L, Arya R. Post-thrombotic syndrome is an independent determinant of health-related quality of life following both first proximal and distal deep vein thrombosis. Haematologica. 2014;99(3):e41-3.
8. Mammen EF. Pathogenesis of venous thrombosis. Chest. 1992;102(6 Suppl):640S-4S.
9. Domeij-Arverud E, Labruto F, Latifi A, Nilsson G, Edman G, Ackermann PW. Intermittent pneumatic compression reduces the risk of deep vein thrombosis during post-operative lower limb immobilisation - a prospective randomised trial of acute ruptures of the Achilles tendon. Bone Joint J 2015;97(B(5)):675-80.
10. Lapidus LJ, Ponzer S, Elvin A, Levander C, Larfars G, Rosfors S, et al. Prolonged thromboprophylaxis with Dalteparin during immobilization after ankle fracture surgery: a randomized placebo-controlled, double-blind study. Acta Orthop. 2007;78(4):528-35.
11. Zee AA, van Lieshout K, van der Heide M, Janssen L, Janzing HM. Low molecular weight heparin for prevention of venous thromboembolism in patients with lower-limb immobilization. Cochrane Database Syst Rev. 2017;8:CD006681.
12. Jorgensen PS, Warming T, Hansen K, Paltved C, Vibeke Berg H, Jensen R, et al. Low molecular weight heparin (Innohep) as thromboprophylaxis in outpatients with a plaster cast: a venografic controlled study. Thrombosis research. 2002;105(6):477-80.
13. Lapidus LJ, Rosfors S, Ponzer S, Levander C, Elvin A, Larfars G, et al. Prolonged thromboprophylaxis with dalteparin after surgical treatment of achilles tendon rupture: a randomized, placebo-controlled study. J Orthop Trauma. 2007;21(1):52-7.
14. Falck-Ytter Y, Francis CW, Johanson NA, Curley C, Dahl OE, Schulman S, et al. Prevention of VTE in orthopedic surgery patients: Antithrombotic Therapy and Prevention of Thrombosis, 9th ed: American College of Chest Physicians Evidence-Based Clinical Practice Guidelines. Chest. 2012;141(2 Suppl):e278S-325S.
15. van Adrichem RA, Nemeth B, Algra A, le Cessie S, Rosendaal FR, Schipper IB, et al. Thromboprophylaxis after Knee Arthroscopy and Lower-Leg Casting. N Engl J Med. 2017;376(6):515-25.
16. Street JT, McGrath M, O'Regan K, Wakai A, McGuinness A, Redmond HP. Thromboprophylaxis using a low molecular weight heparin delays fracture repair. Clin Orthop Relat Res. 2000(381):278-89.
17. Treasure T. Venous thromboembolism: reducing the risk In: Excellence NIfHaC, editor. CG92 ed. London: National Institute for Health and Clinical Excellence; 2010. p. 24-8.
18. utvärdering Sbfmos. Blodpropp - förebyggande, diagnostik och behandling av venös tromboembolism en systematisk kunskapssammanställning [SBU rapport]. Stockholm2002. Available

For peer review only - http://bmjopen.bmj.com/site/about/guidelines.xhtml

from: https://www.sbu.se/sv/publikationer/SBU-utvarderar/blodpropp---forebyggande-diagnostik-och-behandling-av-venos-tromboembolism/.
19. Gee E. The National VTE Exemplar Centres Network response to implementation of updated NICE guidance: venous thromboembolism in over 16s: reducing the risk of hospital-acquired deep vein thrombosis or pulmonary embolism (NG89). Br J Haematol. 2019;186(5):792-3.
20. Barrera LM, Perel P, Ker K, Cirocchi R, Farinella E, Morales Uribe CH. Thromboprophylaxis for trauma patients. Cochrane Database Syst Rev. 2013(3):CD008303.
21. Aufwerber S, Edman G, Gravare Silbernagel K, Ackermann PW. Changes in Tendon Elongation and Muscle Atrophy Over Time After Achilles Tendon Rupture Repair: A Prospective Cohort Study on the Effects of Early Functional Mobilization. Am J Sports Med. 2020;48(13):3296-305.
22. Alim MA, Svedman S, Edman G, Ackermann PW. Procollagen markers in microdialysate can predict patient outcome after Achilles tendon rupture. BMJ Open Sport & Exercise Medicine. 2016;2(1).
23. Chan AW, Tetzlaff JM, Altman DG, Laupacis A, Gotzsche PC, Krleza-Jeric K, et al. SPIRIT 2013 statement: defining standard protocol items for clinical trials. Ann Intern Med. 2013;158(3):200-7.
24. Harris PA, Taylor R, Thielke R, Payne J, Gonzalez N, Conde JG. Research electronic data capture (REDCap)--a metadata-driven methodology and workflow process for providing translational research informatics support. J Biomed Inform. 2009;42(2):377-81.
25. Aufwerber S, Heijne A, Edman G, Gravare Silbernagel K, Ackermann PW. Early mobilization does not reduce the risk of deep venous thrombosis after Achilles tendon rupture: a randomized controlled trial. Knee Surg Sports Traumatol Arthrosc. 2020;28(1):312-9.
26. Devlin NJ, Shah KK, Feng Y, Mulhern B, van Hout B. Valuing health-related quality of life: An EQ-5D-5L value set for England. Health Econ. 2018;27(1):7-22.
27. Roos EM, Brandsson S, Karlsson J. Validation of the foot and ankle outcome score for ankle ligament reconstruction. Foot Ankle Int. 2001;22(10):788-94.
28. Nilsson GM, Eneroth M, Ekdahl CS. The Swedish version of OMAS is a reliable and valid outcome measure for patients with ankle fractures. BMC Musculoskelet Disord. 2013;14:109.
29. Nilsson-Helander K, Thomee R, Silbernagel KG, Thomee P, Faxen E, Eriksson BI, et al. The Achilles tendon Total Rupture Score (ATRS): development and validation. Am J Sports Med. 2007;35(3):421-6.
30. Aufwerber S, Heijne A, Gravare Silbernagel K, Ackermann PW. High Plantar Force Loading After Achilles Tendon Rupture Repair With Early Functional Mobilization. Am J Sports Med. 2019;47(4):894-900.
31. Greve K, Domeij-Arverud E, Labruto F, Edman G, Bring D, Nilsson G, et al. Metabolic activity in early tendon repair can be enhanced by intermittent pneumatic compression. Scand J Med Sci Sports. 2012;22(4):e55-63.
32. Collaboration CT, Dennis M, Sandercock P, Reid J, Graham C, Forbes J, et al. Effectiveness of intermittent pneumatic compression in reduction of risk of deep vein thrombosis in patients who have had a stroke (CLOTS 3): a multicentre randomised controlled trial. Lancet. 2013;382(9891):516-24.
33. Falck-Ytter Y, Francis CW, Johanson NA, Curley C, Dahl OE, Schulman S, et al. Prevention of VTE in orthopedic surgery patients: Antithrombotic Therapy and Prevention of Thrombosis, 9th ed: American College of Chest Physicians Evidence-Based Clinical Practice Guidelines. Chest. 2012;141(2 Suppl):e278S-e325S.
34. Treasure T, Hill J. NICE guidance on reducing the risk of venous thromboembolism in patients admitted to hospital. J R Soc Med. 2010;103(6):210-2.
35. Calder JD, Freeman R, Domeij-Arverud E, van Dijk CN, Ackermann PW. Meta-analysis and suggested guidelines for prevention of venous thromboembolism (VTE) in foot and ankle surgery. Knee Surg Sports Traumatol Arthrosc. 2016;24(4):1409-20.

For peer review only - http://bmjopen.bmj.com/site/about/guidelines.xhtml

36. Batra S, Kurup H, Gul A, Andrew JG. Thromboprophylaxis following cast immobilisation for lower limb injuries--survey of current practice in United Kingdom. Injury. 2006;37(9):813-7.
37. Fox G, Gabbe BJ, Richardson M, Oppy A, Page R, Edwards ER, et al. Twelve-month outcomes following surgical repair of the Achilles tendon. Injury. 2016;47(10):2370-4.
38. Sampson FC, Goodacre S, Kelly AM, Kerr D. How is deep vein thrombosis diagnosed and managed in UK and Australian emergency departments? Emerg Med J. 2005;22(11):780-2.
39. Svedman S, Westin O, Aufwerber S, Edman G, Nilsson-Helander K, Carmont MR, et al. Longer duration of operative time enhances healing metabolites and improves patient outcome after Achilles tendon rupture surgery. Knee Surg Sports Traumatol Arthrosc. 2018;26(7):2011-20.
40. Krutman M, Kuzniec S, Ramacciotti E, Varella AY, Zlotnik M, Teivelis MP, et al. Rediscussing Anticoagulation in Distal Deep Venous Thrombosis. Clin Appl Thromb Hemost. 2016;22(8):772-8.

For peer review only

For peer review only - http://bmjopen.bmj.com/site/about/guidelines.xhtml
